# Supplementary material for: The Limits and Avoidance of Biases in Metagenomic Analyses of Human Fecal Microbiota
Source: Microorganisms. 2020 Dec 9;8(12):1954. doi: 10.3390/microorganisms8121954 (PMC7764459; doi:10.3390/microorganisms8121954)
Supplement: Supplementary file 1 [file microorganisms-08-01954-s001.zip › microorganisms-1008639 suppl final.pdf]

microorganisms

MDPI

Supplementary Materials

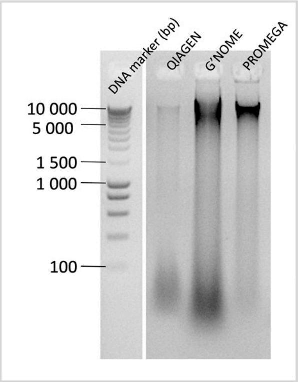

Figure S1. DNA quality according to three fecal DNA extraction methods. Representative electrophoretic profiles of DNA extracted from feces using QIAGEN, G'NOME and PROMEGA methods.

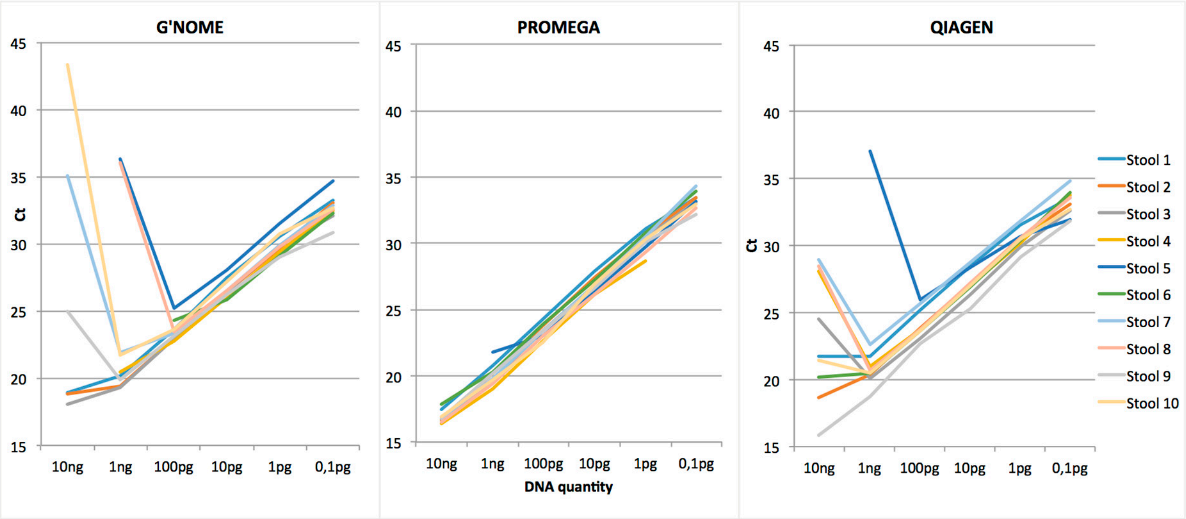

Figure S2. Inhibitory effect according to DNA extraction methods. Linear regression line for G'NOME, PROMEGA and QIAGEN DNA extraction methods. Universal primers and probes were used to detect "All bacteria" by qPCR on a large range of initial DNA quantity.

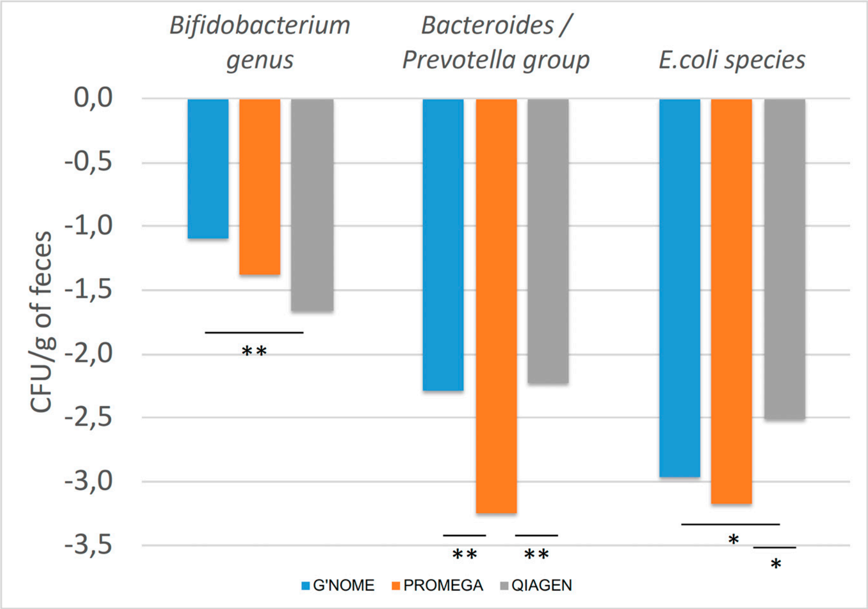

Figure S3. Composition of microbiota regarding dominant bacteria groups from mono-bacterial qPCR detection, according to three different DNA extraction methods. For each bacteria, Log_{10} of CFU/g of feces are normalized by all bacteria amount. Averages ±SEM. *Wilcoxon matched-pairs signed rank test; **p* < 0.05, ***p* < 0.001.

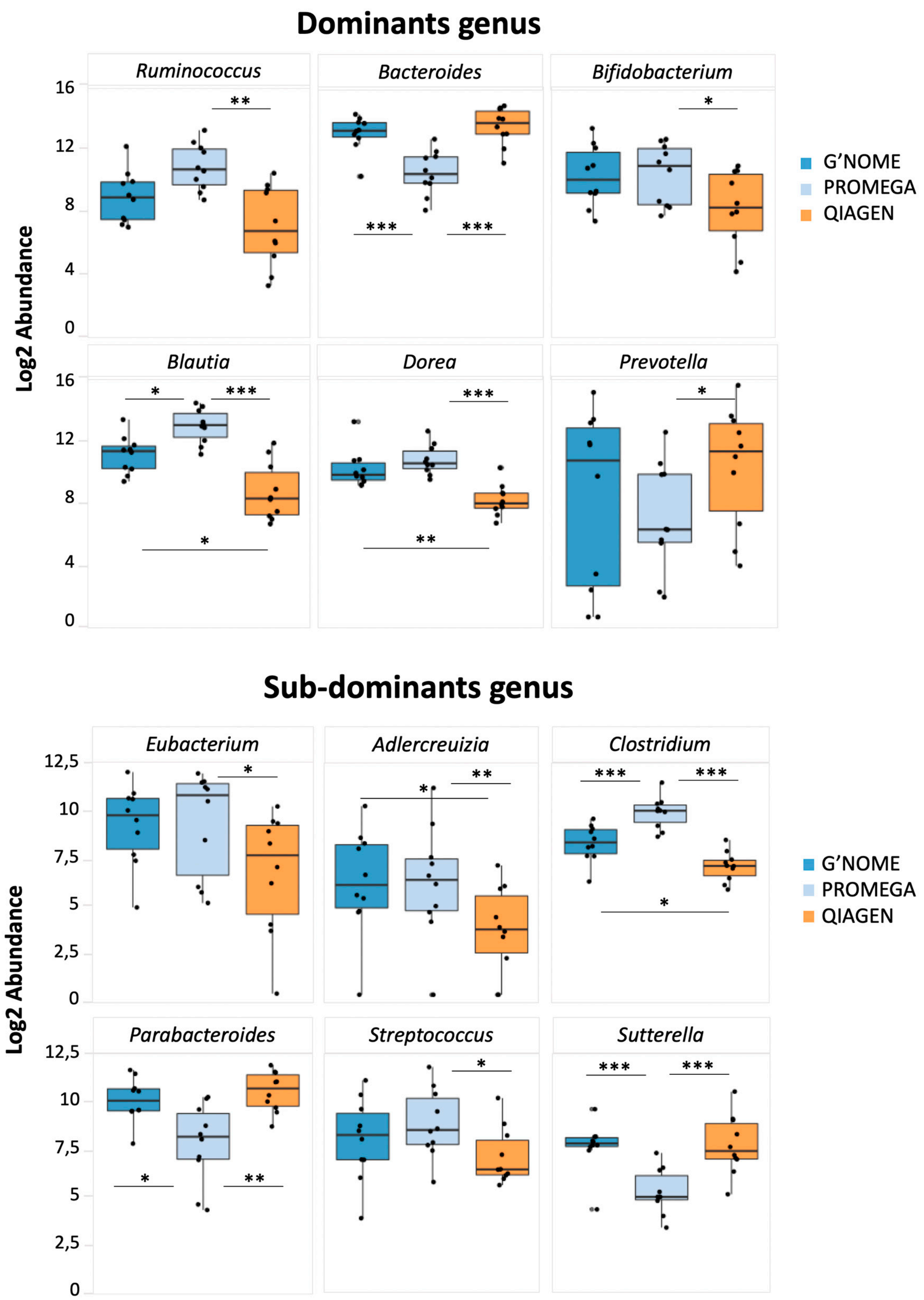

Figure S4. Boxplots depicting changes in the dominant and sub-dominant genus populations in the microbiome of feces identified upon extraction of DNA using three different methods.

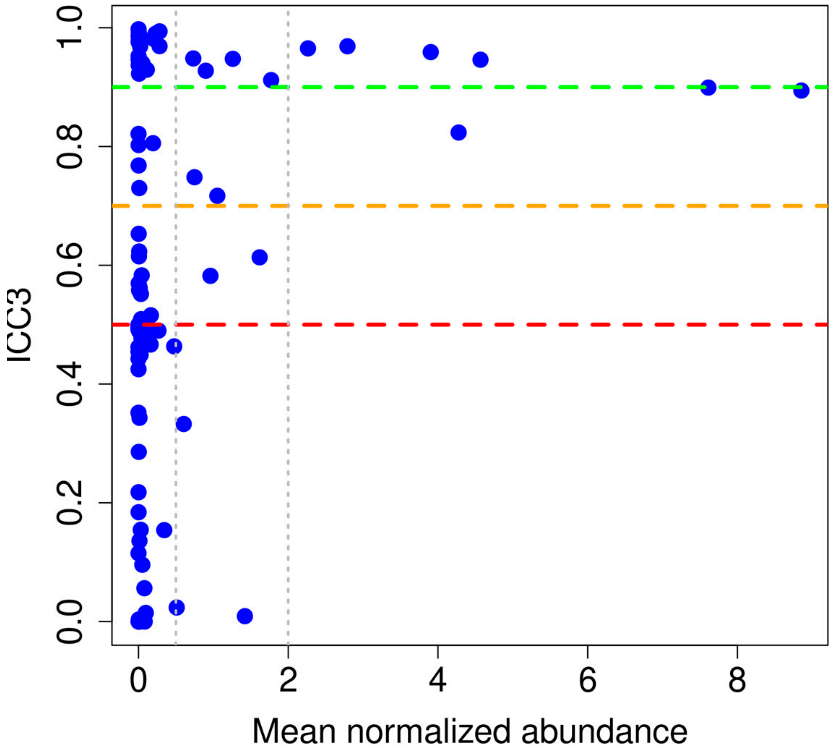

Figure S5. Mean abundance of the 89 commons genera versus ICC (intra-class correlation, ICC3k from the psych (Version 1.9.12.31) package). According to the flowchart proposed [32] ICC estimates and their 95% confident intervals were calculated using the psych package based on a mean-rating (k = 3), absolute agreement, 2-way mixed effects. Horizontal dotted lines: ICC of 0.5 (“red”), 0.70 (“orange”) and 0.9 (“green”), shown as a guideline (given by [32]): ICC < 0.50: poor, 0.50 < ICC < 0.75: moderate, 0.75 < ICC < 0.90: good, ICC > 0.90: excellent. Vertical dotted lines (x-axis: 0.5 and 2) are for visual help.

Table S1: Excel file

Table S2. Group- and species-specific 16S rRNA gene-targeted primers and probes used in this study.

Target organism	Primers and probes	Sequences 5′ 3′	References
	F_Bact 1369	CGG TGA ATA CGT TCC CGG	
**All-bacteria**	R_Prok1492	TAC GGC TAC CTT GTT ACG ACT T	[17]
	P_TM1389F	6FAM-CTT GTA CAC ACC GCC CGT C	
	F_Bifid 09c	CGG GTG AGT AAT GCG TGA CC	
Bifidobacterium genus	R_Bifid 06	TGA TAG GAC GCG ACC CCA	[15]
	P_Bifid	6FAM-CTC CTG GAA ACG GGT G	
	F_Bacter 11	CCT WCG ATG GAT AGG GGT T	[15]
Bacteroides/Prevotella group	R_Bacter 08	CAC GCT ACT TGG CTG GTT CAG	
	P_Bac303	VIC-AAG GTC CCC CAC ATT G	[18]
E. coli species	E.coli F	CAT GCC GCG TGT ATG AAG AA	[19]
	E.coli R	CGG GTA ACG TCA ATG AGC AAA	
