# Supplementary material for: The Limits and Avoidance of Biases in Metagenomic Analyses of Human Fecal Microbiota
Source: Microorganisms. 2020 Dec 9;8(12):1954. doi: 10.3390/microorganisms8121954 (PMC7764459; doi:10.3390/microorganisms8121954)
Supplement: Supplementary file 1 [file microorganisms-08-01954-s001.zip › Suppl-Table-S1_article-16S-supp-tableAbundance-corrected-rev-27-11-2020-filtered.pdf]

filtered

Genus	MOTU	MALT	QIIME1	DADA2	#16S	SORT1
Bacteroides	0.11	4.96	8.83	9.05	3	1
Ruminococcus	0.08	5.25	7.03	7.55	3	2
Clostridium	0.08	0.29	0.54	0.49	3	3
Eubacterium	0.05	0.00	5.21	0.02	3	4
Blautia	0.05	6.21	10.37	9.98	3	5
Prevotella	0.05	0.11	4.15	5.28	3	6
Faecalibacterium	0.04	2.81	5.51	4.51	3	7
Bifidobacterium	0.03	0.98	1.44	1.36	3	8
Streptococcus	0.03	3.24	4.44	4.04	3	9
Roseburia	0.02	2.35	1.69	0.81	3	10
Alistipes	0.02	0.49	0.80	0.96	3	11
Anaerostipes	0.02	2.54	3.17	2.66	3	12
Coprococcus	0.02	0.85	0.71	1.92	3	13
Butyrivibrio	0.01	0.08	0.83	0.52	3	14
Dorea	0.01	0.42	1.09	1.37	3	15
Methanobrevibacter	0.01	0.69	1.08	0.93	3	16
Ruminiclostridium	0.01	0.08	1.44	0.79	3	17
Succinivibrio	0.00	0.24	0.00	0.25	3	18
Holdemanella	0.00	0.14	0.18	0.26	3	19
Parabacteroides	0.00	0.58	0.45	0.41	3	20
Barnesiella	0.00	0.04	0.01	0.06	3	21
Enterococcus	0.00	0.00	0.22	0.17	3	22
Paraprevotella	0.00	0.28	0.29	0.27	3	23
Tyzzerella	0.00	0.06	0.07	0.12	3	24
Staphylococcus	0.00	0.00	0.01	0.01	3	25
Lactobacillus	0.00	1.36	0.19	0.26	3	26
Lactococcus	0.00	0.19	0.23	0.19	3	27
Subdoligranulum	0.00	2.40	2.09	2.30	3	28
Lachnoclostridium	0.00	1.36	2.73	1.23	3	29
Desulfovibrio	0.00	0.26	0.31	0.27	3	30
Lachnospiraceae	0.02	NA	2.87	1.91	2	31
Oscillibacter	0.01	NA	0.02	0.14	2	32
Collinsella	0.01	NA	0.00	0.00	2	33
Phascolarctobacterium	0.01	NA	1.05	1.30	2	34
Dialister	0.01	NA	0.60	0.57	2	35
Akkermansia	0.01	NA	1.05	0.88	2	36
Eggerthella	0.00	NA	0.00	0.00	2	37
Megamonas	0.00	NA	0.39	0.34	2	38
Butyricicoccus	0.00	NA	0.00	0.20	2	39
Odoribacter	0.00	NA	0.09	0.09	2	40
Sutterella	0.00	NA	0.20	0.15	2	41
Megasphaera	0.00	NA	0.66	0.68	2	42
Mitsuokella	0.00	NA	0.17	0.13	2	43
Acidaminococcus	0.00	NA	0.33	0.40	2	44
Parasutterella	0.00	NA	0.00	0.20	2	45
Azospirillum	0.00	NA	0.00	NA	1	46
Enterobacteriaceae	0.01	NA	NA	NA	0	47
Bacteroidales gen. [C Bacteroides/P	0.01	NA	NA	NA	0	48
Bacteria gen. incertae sedis	0.01	NA	NA	NA	0	49
Clostridiales	0.00	NA	NA	NA	0	50
Mycoplasma	0.00	NA	NA	NA	0	51
Intestinibacter	0.00	0.00	0.02	0.27	3	52
Anaerotruncus	0.00	0.39	0.40	0.01	3	53
Bilophila	0.00	0.05	0.07	0.06	3	54

Faecalitalea	0.00	0.01	0.01	0.02	3	55
Actinomyces	0.00	0.01	0.01	0.01	3	56
Erysipelatoclostridium	0.00	0.01	0.06	0.06	3	57
Acinetobacter	0.00	0.00	0.00	0.00	3	58
Citrobacter	0.00	0.02	0.00	0.01	3	59
Fusobacterium	0.00	0.07	0.14	0.12	3	60
Mogibacterium	0.00	0.07	0.08	0.07	3	61
Haemophilus	0.00	0.00	0.00	0.03	3	62
Methanosphaera	0.00	0.01	0.06	0.06	3	63
Leuconostoc	0.00	0.02	0.02	0.02	3	64
Holdemania	0.00	0.00	0.01	0.01	3	65
Anaerobiospirillum	0.00	0.00	0.00	0.00	3	66
Turicibacter	0.00	0.05	0.00	0.06	3	67
Klebsiella	0.00	0.00	0.00	0.02	3	68
Elusimicrobium	0.00	0.00	0.00	0.00	3	69
Faecalicoccus	0.00	0.00	0.00	0.00	3	70
Rothia	0.00	0.01	0.01	0.01	3	71
Succinatimonas	0.00	0.00	0.01	0.01	3	72
Dielma	0.00	0.00	0.00	0.00	3	73
Solobacterium	0.00	0.02	0.03	0.02	3	74
Granulicatella	0.00	0.00	0.00	0.01	3	75
Corynebacterium	0.00	0.00	0.01	0.01	3	76
Alloscardovia	0.00	0.00	0.01	0.01	3	77
Pediococcus	0.00	0.00	0.00	0.00	3	78
Peptostreptococcus	0.00	0.00	0.05	0.04	3	79
Porphyromonas	0.00	0.00	0.00	0.00	3	80
Escherichia	0.00	0.01	0.10	0.97	3	81
Oribacterium	0.00	0.00	0.00	0.00	3	82
Abiotrophia	0.00	0.00	0.00	0.00	3	83
Brevibacterium	0.00	0.00	0.00	0.00	3	84
Pseudomonas	0.00	0.00	0.25	0.00	3	85
Anaerofustis	0.00	0.00	0.01	0.01	3	86
Pyramidobacter	0.00	0.00	0.00	0.00	3	87
Campylobacter	0.00	0.00	0.00	0.00	3	88
Lachnoanaerobaculum	0.00	0.00	0.00	0.00	3	89
Synergistes	0.00	0.00	0.00	0.00	3	90
Weissella	0.00	0.00	0.00	0.00	3	91
Varibaculum	0.00	0.00	0.00	0.00	3	92
Acetobacterium	0.00	0.00	0.00	NA	2	93
Carnobacterium	0.00	0.00	0.00	NA	2	94
Sporosarcina	0.00	0.00	0.00	NA	2	95
Enterobacter	0.00	0.00	0.58	NA	2	96
Ruminococcaceae	0.00	NA	5.54	5.94	2	97
Erysipelotrichaceae	0.00	NA	1.12	0.94	2	98
Parvimonas	0.00	NA	0.12	0.10	2	99
Veillonella	0.00	NA	0.10	0.10	2	100
Flavonifractor	0.00	NA	0.05	0.07	2	101
Butyricimonas	0.00	NA	0.05	0.04	2	102
Terrisporobacter	0.00	NA	0.04	0.06	2	103
Gemella	0.00	NA	0.03	0.03	2	104
Intestinimonas	0.00	NA	0.01	0.13	2	105
Peptoniphilus	0.00	NA	0.01	0.01	2	106
Hungatella	0.00	NA	0.01	0.01	2	107
Raoultella	0.00	NA	0.00	0.00	2	108
Coprobacillus	0.00	NA	0.00	0.00	2	109

Gardnerella	0.00	NA	0.00	0.00	2	110
Atopobium	0.00	NA	0.00	0.00	2	111
Anaerococcus	0.00	NA	0.00	0.00	2	112
Selenomonas	0.00	NA	0.00	0.01	2	113
Olsenella	0.00	NA	0.00	0.00	2	114
Scardovia	0.00	NA	0.00	0.00	2	115
Finegoldia	0.00	NA	0.00	0.00	2	116
Senegalimassilia	0.00	NA	0.00	0.00	2	117
Enterorhabdus	0.00	NA	0.00	0.00	2	118
Bulleidia	0.00	NA	0.00	0.00	2	119
Aggregatibacter	0.00	NA	0.00	0.00	2	120
Adlercreutzia	0.00	NA	0.00	0.00	2	121
Stomatobaculum	0.00	NA	0.00	0.00	2	122
Arcobacter	0.00	NA	0.00	0.00	2	123
Brachyspira	0.00	NA	0.00	0.00	2	124
Pseudoflavonifractor	0.00	NA	NA	0.00	1	125
Merdibacter	0.00	NA	NA	0.00	1	126
Libanicoccus	0.00	NA	NA	0.00	1	127
Mailhella	0.00	NA	NA	0.02	1	128
Lautropia	0.00	NA	NA	0.00	1	129
Serratia	0.00	NA	0.07	NA	1	130
Propionibacterium	0.00	NA	0.00	NA	1	131
Slackia	0.00	NA	0.00	NA	1	132
Hafnia	0.00	NA	0.00	NA	1	133
Microbacterium	0.00	NA	0.00	NA	1	134
Parascardovia	0.00	NA	0.00	NA	1	135
Tropheryma	0.00	NA	0.00	NA	1	136
Macrococcus	0.00	NA	0.00	NA	1	137
Solibacillus	0.00	NA	0.00	NA	1	138
Enorma	0.00	NA	NA	NA	0	139
Porphyromonadaceae	0.00	NA	NA	NA	0	140
Acetobacter	0.00	NA	NA	NA	0	141
Anaeromassilibacillus	0.00	NA	NA	NA	0	142
Clostridiales Family XIII	0.00	NA	NA	NA	0	143
Niameybacter	0.00	NA	NA	NA	0	144
Coraliomargarita	0.00	NA	NA	NA	0	145
Massiliomicrobiota	0.00	NA	NA	NA	0	146
Coriobacteriaceae	0.00	NA	NA	NA	0	147
Actinobaculum	0.00	NA	NA	NA	0	148
Neglecta	0.00	NA	NA	NA	0	149
candidatus Methanomethylophilus	0.00	NA	NA	NA	0	150
Acidiphilium	0.00	NA	NA	NA	0	151
Corallococcus	0.00	NA	NA	NA	0	152
Firmicutes	0.00	NA	NA	NA	0	153
Levyella	0.00	NA	NA	NA	0	154
Tissierellia	0.00	NA	NA	NA	0	155
Bavariicoccus	0.00	NA	NA	NA	0	156
Cryptobacterium	0.00	NA	NA	NA	0	157
Fenollaria	0.00	NA	NA	NA	0	158
Peptostreptococcaceae	0.00	NA	NA	NA	0	159
Helicobacter	0.00	NA	NA	NA	0	160
Acetanaerobacterium	NA	0.00	0.00	0.01	3	161
Alcaligenes	NA	0.00	0.00	0.00	3	162
Alloprevotella	NA	0.21	0.24	0.23	3	163
Anaerofilum	NA	0.01	0.01	0.05	3	164

Bacillus	NA	0.00	0.00	0.00	3	165
Blastocatella	NA	0.00	0.00	0.00	3	166
Bradyrhizobium	NA	0.00	0.00	0.00	3	167
Brevibacillus	NA	0.00	0.00	0.00	3	168
Catenibacterium	NA	0.00	0.27	0.22	3	169
Caulobacter	NA	0.00	0.01	0.01	3	170
Cellulosilyticum	NA	0.00	0.00	0.00	3	171
Christensenella	NA	0.00	0.00	0.89	3	172
Cloacibacillus	NA	0.04	0.07	0.05	3	173
Coprobacter	NA	0.03	0.03	0.04	3	174
Dysgonomonas	NA	0.00	0.00	0.00	3	175
Eisenbergiella	NA	0.03	0.01	0.05	3	176
Filifactor	NA	0.00	0.00	0.00	3	177
Fretibacterium	NA	0.00	0.00	0.00	3	178
Fusicatenibacter	NA	0.65	0.88	0.67	3	179
Johnsonella	NA	0.00	0.00	0.00	3	180
Lachnospira	NA	0.77	0.76	1.63	3	181
Leptotrichia	NA	0.00	0.00	0.00	3	182
Marvinbryantia	NA	0.05	0.85	0.13	3	183
Methylobacillus	NA	0.00	0.00	0.00	3	184
Methylobacterium	NA	0.00	0.01	0.00	3	185
Moryella	NA	0.08	0.01	0.00	3	186
Neisseria	NA	0.00	0.00	0.00	3	187
Oscillospira	NA	0.20	0.01	0.02	3	188
Papillibacter	NA	0.01	0.00	0.00	3	189
Peptococcus	NA	0.00	0.05	0.03	3	190
Rhodopseudomonas	NA	0.00	0.00	0.00	3	191
Robinsoniella	NA	0.00	0.00	0.00	3	192
Sphingomonas	NA	4.69	4.05	4.97	3	193
Sporobacter	NA	0.00	0.00	0.00	3	194
Syntrophococcus	NA	0.00	0.00	0.02	3	195
Treponema	NA	0.00	0.00	0.00	3	196
Variovorax	NA	0.00	0.00	0.00	3	197
Vibrio	NA	0.00	0.00	0.00	3	198
Victivallis	NA	0.01	0.02	0.01	3	199
Desulfotomaculum	NA	0.00	0.00	NA	2	200
Pedobacter	NA	0.00	0.00	NA	2	201
Pseudoxanthomonas	NA	0.00	0.00	NA	2	202
Ethanoligenens	NA	0.00	0.00	NA	2	203
Cedecea	NA	0.00	0.00	NA	2	204
Psychrobacter	NA	0.00	0.00	NA	2	205
Shimwellia	NA	0.00	0.00	NA	2	206
Acetivibrio	NA	0.02	0.00	NA	2	207
Planococcus	NA	0.00	0.00	NA	2	208
Brevundimonas	NA	0.00	0.00	NA	2	209
Mobilitalea	NA	0.00	0.00	NA	2	210
Salmonella	NA	0.00	0.00	NA	2	211
Fastidiosipila	NA	0.00	0.00	NA	2	212
Methanobacterium	NA	0.00	0.00	NA	2	213
Kluyvera	NA	0.00	0.00	NA	2	214
Trichococcus	NA	0.00	0.00	NA	2	215
Anaerosporobacter	NA	0.03	0.00	NA	2	216
Gelria	NA	0.00	0.00	NA	2	217
Acetitomaculum	NA	0.00	0.01	NA	2	218
Cronobacter	NA	0.01	0.01	NA	2	219

Peptoclostridium	NA	0.00	0.12	NA	2	220
Pseudobutyrivibrio	NA	0.01	1.76	NA	2	221
Paeniclostridium	NA	0.00	NA	0.00	2	222
Rosenbergiella	NA	0.00	NA	0.00	2	223
Anaeroplasma	NA	0.00	NA	0.09	2	224
Rikenella	NA	0.00	NA	0.00	2	225
Candidatus_Soleaferrea	NA	0.00	NA	0.01	2	226
Sellimonas	NA	0.02	NA	0.14	2	227
Caproiciproducens	NA	0.04	NA	0.01	2	228
Catenisphaera	NA	0.07	NA	0.13	2	229
Paracoccus	NA	1.77	NA	1.96	2	230
NA	NA	42.27	NA	10.60	2	231
Acidovorax	NA	NA	0.00	0.00	2	232
Actinobacillus	NA	NA	0.03	0.00	2	233
Aerococcus	NA	NA	0.00	0.00	2	234
Aeromonas	NA	NA	0.00	0.00	2	235
Afipia	NA	NA	0.00	0.00	2	236
Allisonella	NA	NA	0.01	0.01	2	237
Asaccharospora	NA	NA	0.00	0.00	2	238
Asteroleplasma	NA	NA	0.00	0.00	2	239
Bdellovibrio	NA	NA	0.00	0.00	2	240
Belnapia	NA	NA	0.00	0.00	2	241
Bergeyella	NA	NA	0.00	0.00	2	242
Brachybacterium	NA	NA	0.00	0.00	2	243
Burkholderia	NA	NA	0.00	0.00	2	244
Catabacter	NA	NA	0.00	0.00	2	245
Chroococcidiopsis	NA	NA	0.00	0.00	2	246
Chryseobacterium	NA	NA	0.00	0.00	2	247
Cloacibacterium	NA	NA	0.00	0.00	2	248
Comamonas	NA	NA	0.00	0.00	2	249
Conchiformibius	NA	NA	0.00	0.00	2	250
Coriobacteriaceae_UCG	NA	NA	0.00	0.00	2	251
Defluviitaleaceae	NA	NA	0.04	0.02	2	252
Delftia	NA	NA	0.00	0.00	2	253
Eikenella	NA	NA	0.00	0.00	2	254
Epulopiscium	NA	NA	0.04	0.03	2	255
Extensimonas	NA	NA	0.00	0.00	2	256
Ezakiella	NA	NA	0.00	0.00	2	257
Fructobacillus	NA	NA	0.00	0.00	2	258
Geodermatophilus	NA	NA	0.01	0.01	2	259
Gordonibacter	NA	NA	0.00	0.00	2	260
Howardella	NA	NA	0.05	0.06	2	261
Hydrogenoanaerobacterium	NA	NA	0.00	0.00	2	262
Hymenobacter	NA	NA	0.00	0.00	2	263
Janibacter	NA	NA	0.00	0.00	2	264
Kocuria	NA	NA	0.00	0.00	2	265
Lactonifactor	NA	NA	0.02	0.00	2	266
Leucobacter	NA	NA	0.00	0.00	2	267
Lysinibacillus	NA	NA	0.00	0.00	2	268
Mannheimia	NA	NA	0.00	0.00	2	269
Micrococcus	NA	NA	0.00	0.00	2	270
Murdochiella	NA	NA	0.00	0.00	2	271
Nesterenkonia	NA	NA	0.00	0.00	2	272
Nocardioides	NA	NA	0.00	0.00	2	273
Novosphingobium	NA	NA	0.00	0.00	2	274

Ottowia	NA	NA	0.00	0.00	2	275
Oxalobacter	NA	NA	0.01	0.01	2	276
Pantoea	NA	NA	0.00	0.00	2	277
Phocaeicola	NA	NA	0.00	0.00	2	278
Phyllobacterium	NA	NA	0.00	0.00	2	279
Prevotellaceae	NA	NA	0.11	0.10	2	280
Pseudoramibacter	NA	NA	0.00	0.00	2	281
Rikenellaceae	NA	NA	0.08	0.05	2	282
Romboutsia	NA	NA	0.58	0.54	2	283
Roseomonas	NA	NA	0.00	0.00	2	284
Rubellimicrobium	NA	NA	0.00	0.00	2	285
Rubrobacter	NA	NA	0.00	0.00	2	286
Shuttleworthia	NA	NA	0.00	0.01	2	287
Spirosoma	NA	NA	0.00	0.00	2	288
Stenotrophomonas	NA	NA	0.00	0.00	2	289
Succiniclasticum	NA	NA	0.00	0.00	2	290
Truepera	NA	NA	0.00	0.00	2	291
Acetatifactor	NA	0.00	NA	NA	1	292
Acholeplasma	NA	0.00	NA	NA	1	293
Emticicia	NA	0.00	NA	NA	1	294
Kosakonia	NA	0.00	NA	NA	1	295
Paraclostridium	NA	0.00	NA	NA	1	296
Sulfurifustis	NA	0.00	NA	NA	1	297
Glutamicibacter	NA	0.00	NA	NA	1	298
Thioprofundum	NA	0.00	NA	NA	1	299
Shigella	NA	0.00	NA	NA	1	300
Cerasicoccus	NA	0.00	NA	NA	1	301
Anaerovorax	NA	0.00	NA	NA	1	302
Sulfitobacter	NA	6.77	NA	NA	1	303
Adhaeribacter	NA	NA	0.00	NA	1	304
Aeribacillus	NA	NA	0.00	NA	1	305
Agromyces	NA	NA	0.00	NA	1	306
Albidovulum	NA	NA	0.00	NA	1	307
Alicycliphilus	NA	NA	0.00	NA	1	308
Alkalibacterium	NA	NA	0.00	NA	1	309
Alloiococcus	NA	NA	0.00	NA	1	310
alpha-proteobacterium	NA	NA	0.00	NA	1	311
Amaricoccus	NA	NA	0.00	NA	1	312
Anaerovibrio	NA	NA	0.00	NA	1	313
Arabidopsis	NA	NA	0.00	NA	1	314
Arsenophonus	NA	NA	0.00	NA	1	315
Artemisia	NA	NA	0.00	NA	1	316
Atopococcus	NA	NA	0.00	NA	1	317
Azoarcus	NA	NA	0.00	NA	1	318
Azorhizobium	NA	NA	0.00	NA	1	319
Bergeriella	NA	NA	0.00	NA	1	320
Bhargavaea	NA	NA	0.00	NA	1	321
Bibersteinia	NA	NA	0.00	NA	1	322
Blastocystis	NA	NA	0.00	NA	1	323
Budvicia	NA	NA	0.00	NA	1	324
Caloramator	NA	NA	0.00	NA	1	325
Centipeda	NA	NA	0.00	NA	1	326
Chryseomicrobium	NA	NA	0.00	NA	1	327
Chungangia	NA	NA	0.00	NA	1	328
Clavibacter	NA	NA	0.00	NA	1	329

Collimonas	NA	NA	0.00	NA	1	330
Curtobacterium	NA	NA	0.00	NA	1	331
Dermacoccus	NA	NA	0.00	NA	1	332
Desulfitibacter	NA	NA	0.00	NA	1	333
Desulfurispora	NA	NA	0.00	NA	1	334
Dolosigranulum	NA	NA	0.00	NA	1	335
Domibacillus	NA	NA	0.00	NA	1	336
Donghicola	NA	NA	0.00	NA	1	337
Dyadobacter	NA	NA	0.00	NA	1	338
Eggerthia	NA	NA	0.00	NA	1	339
Ewingella	NA	NA	0.00	NA	1	340
Fibrobacter	NA	NA	0.00	NA	1	341
Fluviicola	NA	NA	0.00	NA	1	342
Gallicola	NA	NA	0.00	NA	1	343
Geobacillus	NA	NA	0.00	NA	1	344
Helcococcus	NA	NA	0.00	NA	1	345
Hyphomicrobium	NA	NA	0.00	NA	1	346
Jeotgalibacillus	NA	NA	0.00	NA	1	347
Kurthia	NA	NA	0.00	NA	1	348
Legionella	NA	NA	0.00	NA	1	349
Leminorella	NA	NA	0.00	NA	1	350
Leptothrix	NA	NA	0.00	NA	1	351
Listeria	NA	NA	0.00	NA	1	352
Lupinus	NA	NA	0.00	NA	1	353
Luteibacter	NA	NA	0.00	NA	1	354
Marinilactibacillus	NA	NA	0.00	NA	1	355
Methanomassiliicoccus	NA	NA	0.00	NA	1	356
Natranaerovirga	NA	NA	0.00	NA	1	357
Oceanobacillus	NA	NA	0.00	NA	1	358
Ornithinibacillus	NA	NA	0.00	NA	1	359
Paenibacillus	NA	NA	0.00	NA	1	360
Paenisporosarcina	NA	NA	0.00	NA	1	361
Panax	NA	NA	0.00	NA	1	362
Paraeggerthella	NA	NA	0.00	NA	1	363
Paucibacter	NA	NA	0.00	NA	1	364
Paucimonas	NA	NA	0.00	NA	1	365
Pectinatus	NA	NA	0.00	NA	1	366
Pelomonas	NA	NA	0.00	NA	1	367
Photorhabdus	NA	NA	0.00	NA	1	368
Phreatobacter	NA	NA	0.00	NA	1	369
Pleomorphomonas	NA	NA	0.00	NA	1	370
Porphyromonadaceae-bacterium	NA	NA	0.00	NA	1	371
Prochlorococcus	NA	NA	0.00	NA	1	372
Proteiniclasticum	NA	NA	0.00	NA	1	373
Providencia	NA	NA	0.00	NA	1	374
Pseudacidovorax	NA	NA	0.00	NA	1	375
Pseudochrobactrum	NA	NA	0.00	NA	1	376
Rarobacter	NA	NA	0.00	NA	1	377
Rheinheimera	NA	NA	0.00	NA	1	378
Rhizobium	NA	NA	0.00	NA	1	379
Rubrivivax	NA	NA	0.00	NA	1	380
Salinihabitans	NA	NA	0.00	NA	1	381
Shewanella	NA	NA	0.00	NA	1	382
Simplicispira	NA	NA	0.00	NA	1	383
Sphingobacterium	NA	NA	0.00	NA	1	384

Sporacetigenium	NA	NA	0.00	NA	1	385
Stakelama	NA	NA	0.00	NA	1	386
Tatumella	NA	NA	0.00	NA	1	387
Tepidimonas	NA	NA	0.00	NA	1	388
Tetrasphaera	NA	NA	0.00	NA	1	389
Thauera	NA	NA	0.00	NA	1	390
Undibacterium	NA	NA	0.00	NA	1	391
Ureibacillus	NA	NA	0.00	NA	1	392
Virgibacillus	NA	NA	0.00	NA	1	393
Xanthobacter	NA	NA	0.00	NA	1	394
Xanthomonas	NA	NA	0.00	NA	1	395
Xenorhabdus	NA	NA	0.00	NA	1	396
Hespellia	NA	NA	0.00	NA	1	397
unidentified	NA	NA	0.00	NA	1	398
Isoptericola	NA	NA	0.00	NA	1	399
Paenalcaligenes	NA	NA	0.00	NA	1	400
Georgenia	NA	NA	0.00	NA	1	401
Roseovarius	NA	NA	0.00	NA	1	402
Tropicimonas	NA	NA	0.00	NA	1	403
Anoxybacillus	NA	NA	0.00	NA	1	404
Polaromonas	NA	NA	0.00	NA	1	405
Buttiauxella	NA	NA	0.00	NA	1	406
Isobaculum	NA	NA	0.00	NA	1	407
Mesorhizobium	NA	NA	0.00	NA	1	408
Mycobacterium	NA	NA	0.00	NA	1	409
Patulibacter	NA	NA	0.00	NA	1	410
Pseudoclavibacter	NA	NA	0.00	NA	1	411
Segetibacter	NA	NA	0.00	NA	1	412
Sodalis	NA	NA	0.00	NA	1	413
Pragia	NA	NA	0.00	NA	1	414
Schwartzia	NA	NA	0.00	NA	1	415
Kitasatospora	NA	NA	0.00	NA	1	416
Cetobacterium	NA	NA	0.00	NA	1	417
Knoellia	NA	NA	0.00	NA	1	418
Parvibacter	NA	NA	0.00	NA	1	419
Mucispirillum	NA	NA	0.00	NA	1	420
Alishewanella	NA	NA	0.00	NA	1	421
Clostridiales-bacterium	NA	NA	0.00	NA	1	422
Mobiluncus	NA	NA	0.00	NA	1	423
Lacticigenium	NA	NA	0.00	NA	1	424
Thermicanus	NA	NA	0.00	NA	1	425
Aeriscardovia	NA	NA	0.00	NA	1	426
Pseudoscardovia	NA	NA	0.00	NA	1	427
Ralstonia	NA	NA	0.00	NA	1	428
Geminicoccus	NA	NA	0.00	NA	1	429
Negativicoccus	NA	NA	0.00	NA	1	430
Streptomyces	NA	NA	0.00	NA	1	431
Marinococcus	NA	NA	0.00	NA	1	432
Marinomonas	NA	NA	0.00	NA	1	433
Frigoribacterium	NA	NA	0.00	NA	1	434
Mycetocola	NA	NA	0.00	NA	1	435
Roseococcus	NA	NA	0.00	NA	1	436
Solirubrobacter	NA	NA	0.00	NA	1	437
Citreicella	NA	NA	0.00	NA	1	438
Tetragenococcus	NA	NA	0.00	NA	1	439

Oenothera	NA	NA	0.00	NA	1	440
Nitrobacter	NA	NA	0.00	NA	1	441
Phytophthora	NA	NA	0.00	NA	1	442
Musa	NA	NA	0.00	NA	1	443
Bosea	NA	NA	0.00	NA	1	444
Capnocytophaga	NA	NA	0.00	NA	1	445
Marinobacter	NA	NA	0.00	NA	1	446
Niabella	NA	NA	0.00	NA	1	447
Noviherbaspirillum	NA	NA	0.00	NA	1	448
Lysobacter	NA	NA	0.00	NA	1	449
Caryophanon	NA	NA	0.00	NA	1	450
Janthinobacterium	NA	NA	0.00	NA	1	451
Rhodococcus	NA	NA	0.00	NA	1	452
Brochothrix	NA	NA	0.00	NA	1	453
Prunus	NA	NA	0.00	NA	1	454
Ochrobactrum	NA	NA	0.00	NA	1	455
Chlorochromatium	NA	NA	0.00	NA	1	456
Trabulsiella	NA	NA	0.00	NA	1	457
Morganella	NA	NA	0.00	NA	1	458
Facklamia	NA	NA	0.00	NA	1	459
Modestobacter	NA	NA	0.00	NA	1	460
Parasporobacterium	NA	NA	0.00	NA	1	461
Herbaspirillum	NA	NA	0.00	NA	1	462
Lutispora	NA	NA	0.00	NA	1	463
Marmoricola	NA	NA	0.00	NA	1	464
Zea	NA	NA	0.00	NA	1	465
Leifsonia	NA	NA	0.00	NA	1	466
Actinotignum	NA	NA	0.00	NA	1	467
Metascardovia	NA	NA	0.00	NA	1	468
Phenylobacterium	NA	NA	0.00	NA	1	469
Cardiobacterium	NA	NA	0.00	NA	1	470
Ideonella	NA	NA	0.00	NA	1	471
Arthrobacter	NA	NA	0.00	NA	1	472
possible	NA	NA	0.00	NA	1	473
Sphingosinicella	NA	NA	0.00	NA	1	474
Paenirhodobacter	NA	NA	0.00	NA	1	475
Massilia	NA	NA	0.00	NA	1	476
Anaeroglobus	NA	NA	0.00	NA	1	477
Proteus	NA	NA	0.00	NA	1	478
Psychrobacillus	NA	NA	0.00	NA	1	479
Hedyosmum	NA	NA	0.00	NA	1	480
Yersinia	NA	NA	0.00	NA	1	481
Exiguobacterium	NA	NA	0.00	NA	1	482
Eutrema	NA	NA	0.00	NA	1	483
Sphingobium	NA	NA	0.00	NA	1	484
Pasteurella	NA	NA	0.00	NA	1	485
Blastococcus	NA	NA	0.00	NA	1	486
Sphingopyxis	NA	NA	0.00	NA	1	487
Caldicoprobacter	NA	NA	0.00	NA	1	488
Carica	NA	NA	0.00	NA	1	489
Plesiomonas	NA	NA	0.00	NA	1	490
Rhodovulum	NA	NA	0.00	NA	1	491
Vagococcus	NA	NA	0.00	NA	1	492
bacterium	NA	NA	0.00	NA	1	493
Methylovorus	NA	NA	0.00	NA	1	494

Roseobacter	NA	NA	0.00	NA	1	495
Smithella	NA	NA	0.00	NA	1	496
Moraxella	NA	NA	0.00	NA	1	497
Rahnella	NA	NA	0.00	NA	1	498
Microbacter	NA	NA	0.00	NA	1	499
Altererythrobacter	NA	NA	0.00	NA	1	500
Blastomonas	NA	NA	0.00	NA	1	501
Pectobacterium	NA	NA	0.00	NA	1	502
Melissococcus	NA	NA	0.00	NA	1	503
Gemmobacter	NA	NA	0.00	NA	1	504
Flavobacterium	NA	NA	0.00	NA	1	505
Falsirhodobacter	NA	NA	0.00	NA	1	506
Marinovum	NA	NA	0.00	NA	1	507
Rhodobacter	NA	NA	0.00	NA	1	508
Allobaculum	NA	NA	0.00	NA	1	509
Erwinia	NA	NA	0.00	NA	1	510
Sphingomonadaceae-bacterium	NA	NA	0.00	NA	1	511
Planomicrobium	NA	NA	0.01	NA	1	512
Candidatus	NA	NA	0.01	NA	1	513
methanogenic	NA	NA	0.01	NA	1	514
Paracocccus	NA	NA	0.03	NA	1	515
Thalassospira	NA	NA	0.06	NA	1	516
Sarcina	NA	NA	0.08	NA	1	517
	NA	NA	0.24	NA	1	518
Family	NA	NA	0.28	NA	1	519
Christensenellaceae	NA	NA	1.38	NA	1	520
Incertae	NA	NA	1.64	NA	1	521
Defluviimonas	NA	NA	2.05	NA	1	522
28-4	NA	NA	NA	0.03	1	523
Agathobacter	NA	NA	NA	1.63	1	524
Angelakisella	NA	NA	NA	0.02	1	525
Atlantibacter	NA	NA	NA	0.00	1	526
CAG-352	NA	NA	NA	0.21	1	527
CAG-56	NA	NA	NA	0.12	1	528
CAG-873	NA	NA	NA	0.00	1	529
Candidatus_Stoquefichus	NA	NA	NA	0.00	1	530
CENA359	NA	NA	NA	0.00	1	531
CHKCI002	NA	NA	NA	0.00	1	532
Cuneatibacter	NA	NA	NA	0.00	1	533
DTU089	NA	NA	NA	0.01	1	534
Enhydrobacter	NA	NA	NA	0.00	1	535
F0332	NA	NA	NA	0.00	1	536
Family_XIII_AD3011_group	NA	NA	NA	0.24	1	537
Family_XIII_UCG-001	NA	NA	NA	0.04	1	538
Flavisolibacter	NA	NA	NA	0.00	1	539
Fournierella	NA	NA	NA	0.03	1	540
GCA-900066225	NA	NA	NA	0.04	1	541
GCA-900066575	NA	NA	NA	0.05	1	542
GCA-900066755	NA	NA	NA	0.00	1	543
Hafnia-Obesumbacterium	NA	NA	NA	0.05	1	544
Harryflintia	NA	NA	NA	0.00	1	545
Herbinix	NA	NA	NA	0.00	1	546
Lawsonella	NA	NA	NA	0.00	1	547
Microvirga	NA	NA	NA	0.00	1	548
Mucilaginibacter	NA	NA	NA	0.00	1	549

Negativibacillus	NA	NA	NA	0.11	1	550
Pantanalinema_CENA516	NA	NA	NA	0.00	1	551
Paraherbaspirillum	NA	NA	NA	0.00	1	552
Pelospora	NA	NA	NA	0.00	1	553
Phocea	NA	NA	NA	0.00	1	554
PMMR1	NA	NA	NA	0.00	1	555
Pygmaiobacter	NA	NA	NA	0.00	1	556
Raoultibacter	NA	NA	NA	0.00	1	557
RB41	NA	NA	NA	0.00	1	558
Rubrivirga	NA	NA	NA	0.00	1	559
Sanguibacteroides	NA	NA	NA	0.00	1	560
Scytonema_UTEX_2349	NA	NA	NA	0.00	1	561
Sphingoaurantiacus	NA	NA	NA	0.00	1	562
UBA1819	NA	NA	NA	0.10	1	563
UC5-1-2E3	NA	NA	NA	0.00	1	564
