# Supplementary material for: The Limits and Avoidance of Biases in Metagenomic Analyses of Human Fecal Microbiota
Source: Microorganisms. 2020 Dec 9;8(12):1954. doi: 10.3390/microorganisms8121954 (PMC7764459; doi:10.3390/microorganisms8121954)
Supplement: Supplementary file 1 [file microorganisms-08-01954-s001.zip › Suppl-Table-S1_article-16S-supp-tableAbundance-corrected-rev-27-11-2020-FlashAndTrim.pdf]

File	W	Q	NbReads	NbSurvived	%Survived	Flash-Total-pairs	Flash-Combined-pairs	Flash-Combined
FR-026	5	20	101177	100834	99.66	100834	91072	90.32
FR-027	5	20	229626	228953	99.71	228953	207936	90.82
FR-030	5	20	117396	116894	99.57	116894	107738	92.17
FR-039	5	20	105952	105525	99.6	105525	95468	90.47
FR-040	5	20	103705	103094	99.41	103094	91805	89.05
FR-051	5	20	110455	110167	99.74	110167	100891	91.58
FR-052	5	20	96288	95936	99.63	95936	86837	90.52
FR-053	5	20	120882	120442	99.64	120442	110186	91.48
FR-054	5	20	120111	119594	99.57	119594	109848	91.85
FR-060	5	20	216451	215356	99.49	215356	196515	91.25
FR-105	5	20	117131	115940	98.98	115940	104448	90.09
FR-110	5	20	313848	312150	99.46	312150	282740	90.58
FR-113	5	20	119250	118840	99.66	118840	108186	91.04
FR-115	5	20	92364	91905	99.5	91905	82828	90.12
FR-116	5	20	95000	94687	99.67	94687	85030	89.80
FR-125	5	20	111076	110428	99.42	110428	97932	88.68
FR-129	5	20	105518	104975	99.49	104975	92970	88.56
FR-132	5	20	126548	126284	99.79	126284	114881	90.97
FR-139	5	20	139807	139405	99.71	139405	119993	86.08
FR-142	5	20	83525	83082	99.47	83082	73811	88.84
FR-152	5	20	120006	119704	99.75	119704	109704	91.65
FR-156	5	20	81391	80955	99.46	80955	73192	90.41
FR-161	5	20	99921	99442	99.52	99442	90266	90.77
FR-162	5	20	116773	116376	99.66	116376	107420	92.30
FR-166	5	20	81388	80860	99.35	80860	69135	85.50
FR-169	5	20	64567	64042	99.19	64042	56604	88.39
FR-170	5	20	414692	412328	99.43	412328	360128	87.34
FR-173	5	20	82000	81459	99.34	81459	73504	90.23
FR-187	5	20	113483	113143	99.7	113143	104484	92.35
FR-191	5	20	106328	105846	99.55	105846	97639	92.25
FR-194	5	20	828967	824659	99.48	824659	741391	89.90
FR-196	5	20	889879	885375	99.49	885375	767150	86.65
FR-198	5	20	642104	640144	99.69	640144	588031	91.86
FR-200	5	20	760950	757674	99.57	757674	679690	89.71

FR-208	5	20	637951	634570	99.47	634570	578927	91.23
FR-211	5	20	702839	700209	99.63	700209	623900	89.10
FR-212	5	20	703194	700037	99.55	700037	636289	90.89
FR-213	5	20	925929	921891	99.56	921891	841400	91.27
FR-214	5	20	707319	704595	99.61	704595	639835	90.81
FR-215	5	20	708115	703953	99.41	703953	637027	90.49
FR-218	5	20	891454	888174	99.63	888174	812366	91.46
FR-221	5	20	1034746	1030153	99.56	1030153	906846	88.03
FR-223	5	20	836386	833709	99.68	833709	746784	89.57
FR-229	5	20	695644	692846	99.6	692846	633180	91.39
FR-241	5	20	744954	740619	99.42	740619	671640	90.69
FR-276	5	20	359086	357340	99.51	357340	327185	91.56
FR-281	5	20	692124	689231	99.58	689231	618732	89.77
FR-294	5	20	405390	403535	99.54	403535	357918	88.70
FR-298	5	20	308208	306736	99.52	306736	283712	92.49
FR-302	5	20	279652	278418	99.56	278418	254703	91.48
FR-305	5	20	238952	237892	99.56	237892	218449	91.83
FR-312	5	20	327031	325507	99.53	325507	299829	92.11
FR-316	5	20	590761	587448	99.44	587448	535952	91.23
FR-328	5	20	305581	302544	99.01	302544	273566	90.42
FR-344	5	20	632969	629607	99.47	629607	569226	90.41
FR-349	5	20	302407	301456	99.69	301456	275231	91.30
FR-390	5	20	313187	311307	99.4	311307	285351	91.66
FR-393	5	20	411829	409780	99.5	409780	369651	90.21
FR-399	5	20	346495	344459	99.41	344459	308842	89.66
FR-400	5	20	381851	379701	99.44	379701	334989	88.22
FR-414	5	20	561757	559892	99.67	559892	489644	87.45
FR-419	5	20	254371	253297	99.58	253297	223605	88.28
FR-430	5	20	262129	261318	99.69	261318	228288	87.36
FR-449	5	20	371606	369294	99.38	369294	317141	85.88
FR-450	5	20	337873	336517	99.6	336517	297420	88.38
FR-451	5	20	308654	307279	99.55	307279	270056	87.89
FR-459	5	20	334489	333180	99.61	333180	294876	88.50
FR-460	5	20	386935	385493	99.63	385493	351338	91.14
FR-473	5	20	344015	342599	99.59	342599	306236	89.39

FR-474	5	20	695167	691421	99.46	691421	614228	88.84
FR-496	5	20	640168	636433	99.42	636433	557839	87.65
FR-500	5	20	272876	271520	99.5	271520	247142	91.02
FR-503	5	20	309172	307732	99.53	307732	267656	86.98
FR-505	5	20	394425	392147	99.42	392147	340393	86.80
FR-506	5	20	440769	438542	99.49	438542	378672	86.35
FR-507	5	20	379853	378209	99.57	378209	342287	90.50
FR-510	5	20	303120	302262	99.72	302262	277614	91.85
FR-539	5	20	325632	323852	99.45	323852	272772	84.23
FR-542	5	20	430463	428926	99.64	428926	379028	88.37
FR-548	5	20	420405	418877	99.64	418877	357154	85.26
FR-551	5	20	201131	199961	99.42	199961	180182	90.11
FR-552	5	20	417077	415207	99.55	415207	363670	87.59
FR-558	5	20	365817	364362	99.6	364362	337004	92.49
FR-568	5	20	315750	314465	99.59	314465	293433	93.31
FR-596	5	20	406889	405103	99.56	405103	358422	88.48
FR-606	5	20	208980	207614	99.35	207614	182728	88.01
FR-617	5	20	308471	306823	99.47	306823	273670	89.19
FR-626	5	20	395113	393980	99.71	393980	351116	89.12
FR-628	5	20	289647	287769	99.35	287769	250726	87.13
FR-643	5	20	337821	335717	99.38	335717	293317	87.37
FR-654	5	20	449026	447355	99.63	447355	398029	88.97
FR-664	5	20	312332	310849	99.53	310849	266661	85.78
FR-666	5	20	324381	322171	99.32	322171	280302	87.00
FR-667	5	20	423828	421721	99.5	421721	373366	88.53
FR-672	5	20	546370	543182	99.42	543182	471662	86.83
FR-682	5	20	391101	389743	99.65	389743	348823	89.50
FR-684	5	20	453304	450400	99.36	450400	396137	87.95
FR-696	5	20	328576	326408	99.34	326408	294898	90.35
FR-716	5	20	362160	360290	99.48	360290	319443	88.66
FR-719	5	20	484767	482121	99.45	482121	428717	88.92
FR-721	5	20	278797	276708	99.25	276708	251875	91.03
FR-722	5	20	168548	167536	99.4	167536	149603	89.30
FR-723	5	20	464344	461332	99.35	461332	394771	85.57
FR-726	5	20	424684	422602	99.51	422602	385413	91.20

FR-728	5	20	346529	345171	99.61	345171	321504	93.14
FR-730	5	20	358698	356825	99.48	356825	315537	88.43
FR-734	5	20	394641	392670	99.5	392670	341126	86.87
FR-751	5	20	561936	559063	99.49	559063	512390	91.65
FR-759	5	20	357113	355028	99.42	355028	315221	88.79
FR-767	5	20	450201	447522	99.4	447522	387077	86.49
FR-768	5	20	485999	483607	99.51	483607	421645	87.19
FR-770	5	20	387129	385385	99.55	385385	337668	87.62
FR-772	5	20	306581	305062	99.5	305062	268641	88.06
FR-780	5	20	303892	301985	99.37	301985	261880	86.72
FR-783	5	20	312544	311394	99.63	311394	281569	90.42
FR-788	5	20	437661	435228	99.44	435228	387111	88.94
FR-790	5	20	353576	352446	99.68	352446	327472	92.91
FR-792	5	20	339311	337680	99.52	337680	307296	91.00
FR-812	5	20	324908	323517	99.57	323517	296163	91.54
FR-817	5	20	318309	316653	99.48	316653	288765	91.19
FR-820	5	20	326352	324735	99.5	324735	292905	90.20
FR-824	5	20	414039	412468	99.62	412468	379341	91.97
FR-825	5	20	272364	271106	99.54	271106	251863	92.90
FR-826	5	20	417195	415053	99.49	415053	367797	88.61
FR-827	5	20	312568	310974	99.49	310974	285312	91.75
FR-828	5	20	415942	413367	99.38	413367	367219	88.84
FR-829	5	20	372153	370821	99.64	370821	338097	91.18
FR-830	5	20	299504	298044	99.51	298044	276096	92.64
FR-835	5	20	274513	273075	99.48	273075	245873	90.04

Trim_NbReads	TrimQ20-Survived	TrimQ20-%Survived	TrimQ30-NbReads	TrimQ30-Survived	TrimQ30-%Survived
91072	88387	97.05	91072	87442	96.01
207936	201136	96.73	207936	198997	95.7
107738	103662	96.22	107738	102291	94.94
95468	92100	96.47	95468	90733	95.04
91805	88336	96.22	91805	87226	95.01
100891	97754	96.89	100891	96713	95.86
86837	83944	96.67	86837	83146	95.75
110186	106654	96.79	110186	105467	95.72
109848	105801	96.32	109848	104557	95.18
196515	190100	96.74	196515	188117	95.73
104448	100736	96.45	104448	99619	95.38
282740	273530	96.74	282740	270274	95.59
108186	104997	97.05	108186	103977	96.11
82828	80362	97.02	82828	79708	96.23
85030	82154	96.62	85030	81323	95.64
97932	94719	96.72	97932	93800	95.78
92970	89804	96.59	92970	89153	95.89
114881	111325	96.9	114881	110226	95.95
119993	117162	97.64	119993	115495	96.25
73811	71610	97.02	73811	71164	96.41
109704	106378	96.97	109704	105397	96.07
73192	70684	96.57	73192	69931	95.54
90266	87561	97	90266	86932	96.31
107420	103619	96.46	107420	102393	95.32
69135	66778	96.59	69135	65996	95.46
56604	54733	96.69	56604	54174	95.71
360128	344131	95.56	360128	340127	94.45
73504	71121	96.76	73504	70416	95.8
104484	101170	96.83	104484	100174	95.87
97639	94630	96.92	97639	93826	96.09
741391	717974	96.84	741391	711063	95.91
767150	743504	96.92	767150	735876	95.92
588031	567122	96.44	588031	556971	94.72
679690	658007	96.81	679690	647881	95.32

578927	552822	95.49	578927	544204	94
623900	604014	96.81	623900	597681	95.8
636289	614616	96.59	636289	608204	95.59
841400	812675	96.59	841400	803790	95.53
639835	618084	96.6	639835	611602	95.59
637027	614910	96.53	637027	610536	95.84
812366	785532	96.7	812366	776780	95.62
906846	875859	96.58	906846	864545	95.34
746784	721437	96.61	746784	710790	95.18
633180	611944	96.65	633180	605443	95.62
671640	647227	96.37	671640	639825	95.26
327185	315409	96.4	327185	311869	95.32
618732	588239	95.07	618732	579259	93.62
357918	343674	96.02	357918	338637	94.61
283712	271797	95.8	283712	268212	94.54
254703	243276	95.51	254703	239743	94.13
218449	211846	96.98	218449	209386	95.85
299829	287404	95.86	299829	283470	94.54
535952	514921	96.08	535952	508820	94.94
273566	259273	94.78	273566	255197	93.29
569226	541556	95.14	569226	532166	93.49
275231	265409	96.43	275231	262074	95.22
285351	275582	96.58	285351	272224	95.4
369651	354251	95.83	369651	349760	94.62
308842	295476	95.67	308842	291343	94.33
334989	321931	96.1	334989	318394	95.05
489644	469838	95.96	489644	463018	94.56
223605	213829	95.63	223605	210855	94.3
228288	220742	96.69	228288	219017	95.94
317141	299644	94.48	317141	294567	92.88
297420	284626	95.7	297420	281100	94.51
270056	256181	94.86	270056	251185	93.01
294876	281304	95.4	294876	277395	94.07
351338	333818	95.01	351338	327849	93.31
306236	292830	95.62	306236	288928	94.35

614228	587904	95.71	614228	578968	94.26
557839	533364	95.61	557839	525096	94.13
247142	236165	95.56	247142	233178	94.35
267656	256130	95.69	267656	251812	94.08
340393	321301	94.39	340393	315367	92.65
378672	360145	95.11	378672	355348	93.84
342287	330402	96.53	342287	326844	95.49
277614	266855	96.12	277614	263188	94.8
272772	262093	96.09	272772	257703	94.48
379028	361344	95.33	379028	355813	93.88
357154	340520	95.34	357154	334261	93.59
180182	170538	94.65	180182	167685	93.06
363670	348553	95.84	363670	344320	94.68
337004	325273	96.52	337004	321894	95.52
293433	282010	96.11	293433	278768	95
358422	341975	95.41	358422	337393	94.13
182728	174505	95.5	182728	172485	94.39
273670	262416	95.89	273670	260079	95.03
351116	338530	96.42	351116	334559	95.28
250726	239728	95.61	250726	236854	94.47
293317	279680	95.35	293317	276194	94.16
398029	380546	95.61	398029	375420	94.32
266661	255252	95.72	266661	248497	93.19
280302	268485	95.78	280302	265913	94.87
373366	357867	95.85	373366	353295	94.62
471662	451189	95.66	471662	446494	94.66
348823	334573	95.91	348823	329835	94.56
396137	379850	95.89	396137	375865	94.88
294898	282074	95.65	294898	279823	94.89
319443	304163	95.22	319443	300157	93.96
428717	409371	95.49	428717	404236	94.29
251875	238377	94.64	251875	234586	93.14
149603	142729	95.41	149603	140644	94.01
394771	376903	95.47	394771	372091	94.25
385413	368177	95.53	385413	363679	94.36

321504	310720	96.65	321504	307757	95.72
315537	302767	95.95	315537	299244	94.84
341126	325826	95.51	341126	321302	94.19
512390	494293	96.47	512390	487394	95.12
315221	306176	97.13	315221	302539	95.98
387077	364115	94.07	387077	357826	92.44
421645	403744	95.75	421645	397519	94.28
337668	319849	94.72	337668	314514	93.14
268641	254726	94.82	268641	250843	93.37
261880	249901	95.43	261880	246631	94.18
281569	271256	96.34	281569	267617	95.04
387111	369472	95.44	387111	362769	93.71
327472	312449	95.41	327472	308046	94.07
307296	293154	95.4	307296	289267	94.13
296163	283549	95.74	296163	279273	94.3
288765	277499	96.1	288765	274203	94.96
292905	284219	97.03	292905	280518	95.77
379341	362994	95.69	379341	358693	94.56
251863	238670	94.76	251863	234864	93.25
367797	353171	96.02	367797	348558	94.77
285312	273342	95.8	285312	269618	94.5
367219	349552	95.19	367219	344786	93.89
338097	323742	95.75	338097	319219	94.42
276096	266010	96.35	276096	263276	95.36
245873	237439	96.57	245873	234411	95.34
