# Supplementary material for: The Limits and Avoidance of Biases in Metagenomic Analyses of Human Fecal Microbiota
Source: Microorganisms. 2020 Dec 9;8(12):1954. doi: 10.3390/microorganisms8121954 (PMC7764459; doi:10.3390/microorganisms8121954)
Supplement: Supplementary file 1 [file microorganisms-08-01954-s001.zip › Suppl-Table-S1_article-16S-supp-tableAbundance-corrected-rev-27-11-2020-raw-results.pdf]

| Genus | MOTU | MALT | QIIME1 | DADA2 | #16S |
|---|---|---|---|---|---|
| g__Abiotrophia | 0.00 | 0.00 | 0.00 | 0.00 | 3 |
| g__Acetivibrio | NA | 0.02 | 0.00 | NA | 2 |
| g__Acetobacter | 0.00 | NA | NA | NA | 0 |
| g__Acetobacterium | 0.00 | 0.00 | 0.00 | NA | 2 |
| g__Acholeplasma | NA | 0.00 | NA | NA | 1 |
| g__Acidaminococcus | 0.00 | NA | 0.33 | 0.40 | 2 |
| g__Acidiphilium | 0.00 | NA | NA | NA | 0 |
| g__Acidovorax | NA | NA | 0.00 | 0.00 | 2 |
| g__Acinetobacter | 0.00 | 0.00 | 0.00 | 0.00 | 3 |
| g__Actinobacillus | NA | NA | 0.03 | 0.00 | 2 |
| g__Actinobaculum | 0.00 | NA | NA | NA | 0 |
| g__Actinomyces | 0.00 | 0.01 | 0.01 | 0.01 | 3 |
| g__Actinotignum | NA | NA | 0.00 | NA | 1 |
| g__Adhaeribacter | NA | NA | 0.00 | NA | 1 |
| g__Adlercreutzia | 0.00 | NA | 0.00 | 0.00 | 2 |
| g__Aerococcus | NA | NA | 0.00 | 0.00 | 2 |
| g__Aeromonas | NA | NA | 0.00 | 0.00 | 2 |
| g__Afipia | NA | NA | 0.00 | 0.00 | 2 |
| g__Aggregatibacter | 0.00 | NA | 0.00 | 0.00 | 2 |
| g__Agromyces | NA | NA | 0.00 | NA | 1 |
| g__Akkermansia | 0.01 | NA | 1.05 | 0.88 | 2 |
| g__Alcaligenes | NA | 0.00 | 0.00 | 0.00 | 3 |
| g__Alicycliphilus | NA | NA | 0.00 | NA | 1 |
| g__Alishewanella | NA | NA | 0.00 | NA | 1 |
| g__Alistipes | 0.02 | 0.49 | 0.80 | 0.96 | 3 |
| g__Alkalibacterium | NA | NA | 0.00 | NA | 1 |
| g__Allobaculum | NA | NA | 0.00 | NA | 1 |
| g__Alloiococcus | NA | NA | 0.00 | NA | 1 |
| g__Alloprevotella | NA | 0.21 | 0.24 | 0.23 | 3 |
| g__Alloscardovia | 0.00 | 0.00 | 0.01 | 0.01 | 3 |
| g__Anaerobiospirillum | 0.00 | 0.00 | 0.00 | 0.00 | 3 |
| g__Anaerococcus | 0.00 | NA | 0.00 | 0.00 | 2 |
| g__Anaerofustis | 0.00 | 0.00 | 0.01 | 0.01 | 3 |
| g__Anaeroglobus | NA | NA | 0.00 | NA | 1 |
| g__Anaeromassilibacillus | 0.00 | NA | NA | NA | 0 |
| g__Anaerostipes | 0.02 | 2.54 | 3.17 | 2.66 | 3 |
| g__Anaerotruncus | 0.00 | 0.39 | 0.40 | 0.01 | 3 |
| g__Anaerovibrio | NA | NA | 0.00 | NA | 1 |
| g__Anaerovorax | NA | 0.00 | NA | NA | 1 |
| g__Anoxybacillus | NA | NA | 0.00 | NA | 1 |
| g__Arcobacter | 0.00 | NA | 0.00 | 0.00 | 2 |
| g__Arsenophonus | NA | NA | 0.00 | NA | 1 |
| g__Arthrobacter | NA | NA | 0.00 | NA | 1 |
| g__Atopobium | 0.00 | NA | 0.00 | 0.00 | 2 |
| g__Atopococcus | NA | NA | 0.00 | NA | 1 |
| g__Azoarcus | NA | NA | 0.00 | NA | 1 |
| g__Azorhizobium | NA | NA | 0.00 | NA | 1 |
| g__Azospirillum | 0.00 | NA | 0.00 | NA | 1 |
| g__Bacillus | NA | 0.00 | 0.00 | 0.00 | 3 |
| g__Bacteria gen. incertae sedis | 0.01 | NA | NA | NA | 0 |
| g__Bacteroidales gen. [C Bacteroides/Porphyromonas/Parabacteroides] | 0.01 | NA | NA | NA | 0 |
| g__Bacteroides | 0.11 | 4.96 | 8.83 | 9.05 | 3 |

| | | | | | |
|---|---|---|---|---|---|
| g__Barnesiella | 0.00 | 0.04 | 0.01 | 0.06 | 3 |
| g__Bavariicoccus | 0.00 | NA | NA | NA | 0 |
| g__Bdellovibrio | NA | NA | 0.00 | 0.00 | 2 |
| g__Belnapia | NA | NA | 0.00 | 0.00 | 2 |
| g__Bergeyella | NA | NA | 0.00 | 0.00 | 2 |
| g__Bhargavaea | NA | NA | 0.00 | NA | 1 |
| g__Bibersteinia | NA | NA | 0.00 | NA | 1 |
| g__Bifidobacterium | 0.03 | 0.98 | 1.44 | 1.36 | 3 |
| g__Bilophila | 0.00 | 0.05 | 0.07 | 0.06 | 3 |
| g__Blastococcus | NA | NA | 0.00 | NA | 1 |
| g__Blastomonas | NA | NA | 0.00 | NA | 1 |
| g__Blautia | 0.05 | 6.21 | 10.37 | 9.98 | 3 |
| g__Bosea | NA | NA | 0.00 | NA | 1 |
| g__Brachybacterium | NA | NA | 0.00 | 0.00 | 2 |
| g__Brachyspira | 0.00 | NA | 0.00 | 0.00 | 2 |
| g__Bradyrhizobium | NA | 0.00 | 0.00 | 0.00 | 3 |
| g__Brevibacillus | NA | 0.00 | 0.00 | 0.00 | 3 |
| g__Brevibacterium | 0.00 | 0.00 | 0.00 | 0.00 | 3 |
| g__Brevundimonas | NA | 0.00 | 0.00 | NA | 2 |
| g__Brochothrix | NA | NA | 0.00 | NA | 1 |
| g__Budvicia | NA | NA | 0.00 | NA | 1 |
| g__Bulleidia | 0.00 | NA | 0.00 | 0.00 | 2 |
| g__Burkholderia | NA | NA | 0.00 | 0.00 | 2 |
| g__Buttiauxella | NA | NA | 0.00 | NA | 1 |
| g__Butyricicoccus | 0.00 | NA | 0.00 | 0.20 | 2 |
| g__Butyricimonas | 0.00 | NA | 0.05 | 0.04 | 2 |
| g__Butyrivibrio | 0.01 | 0.08 | 0.83 | 0.52 | 3 |
| g__Caldicoprobacter | NA | NA | 0.00 | NA | 1 |
| g__Caloramator | NA | NA | 0.00 | NA | 1 |
| g__Campylobacter | 0.00 | 0.00 | 0.00 | 0.00 | 3 |
| g__candidatus Methanomethylophilus | 0.00 | NA | NA | NA | 0 |
| g__Capnocytophaga | NA | NA | 0.00 | NA | 1 |
| g__Cardiobacterium | NA | NA | 0.00 | NA | 1 |
| g__Carnobacterium | 0.00 | 0.00 | 0.00 | NA | 2 |
| g__Caulobacter | NA | 0.00 | 0.01 | 0.01 | 3 |
| g__Cedecea | NA | 0.00 | 0.00 | NA | 2 |
| g__Cellulosilyticum | NA | 0.00 | 0.00 | 0.00 | 3 |
| g__Centipeda | NA | NA | 0.00 | NA | 1 |
| g__Cetobacterium | NA | NA | 0.00 | NA | 1 |
| g__Chroococcidiopsis | NA | NA | 0.00 | 0.00 | 2 |
| g__Chryseobacterium | NA | NA | 0.00 | 0.00 | 2 |
| g__Citreicella | NA | NA | 0.00 | NA | 1 |
| g__Citrobacter | 0.00 | 0.02 | 0.00 | 0.01 | 3 |
| g__Clavibacter | NA | NA | 0.00 | NA | 1 |
| g__Clostridiales | 0.00 | NA | NA | NA | 0 |
| g__Clostridiales Family XIII | 0.00 | NA | NA | NA | 0 |
| g__Clostridium | 0.08 | 0.29 | 0.54 | 0.49 | 3 |
| g__Collimonas | NA | NA | 0.00 | NA | 1 |
| g__Collinsella | 0.01 | NA | 0.00 | 0.00 | 2 |
| g__Comamonas | NA | NA | 0.00 | 0.00 | 2 |
| g__Conchiformibius | NA | NA | 0.00 | 0.00 | 2 |
| g__Coprobacillus | 0.00 | NA | 0.00 | 0.00 | 2 |
| g__Coprococcus | 0.02 | 0.85 | 0.71 | 1.92 | 3 |
| g__Coraliomargarita | 0.00 | NA | NA | NA | 0 |
| g__Corallococcus | 0.00 | NA | NA | NA | 0 |

| | | | | | |
|---|---|---|---|---|---|
| g__Coriobacteriaceae | 0.00 | NA | NA | NA | 0 |
| g__Corynebacterium | 0.00 | 0.00 | 0.01 | 0.01 | 3 |
| g__Cronobacter | NA | 0.01 | 0.01 | NA | 2 |
| g__Cryptobacterium | 0.00 | NA | NA | NA | 0 |
| g__Curtobacterium | NA | NA | 0.00 | NA | 1 |
| g__Defluviimonas | NA | NA | 2.05 | NA | 1 |
| g__Delftia | NA | NA | 0.00 | 0.00 | 2 |
| g__Dermacoccus | NA | NA | 0.00 | NA | 1 |
| g__Desulfitibacter | NA | NA | 0.00 | NA | 1 |
| g__Desulfotomaculum | NA | 0.00 | 0.00 | NA | 2 |
| g__Desulfovibrio | 0.00 | 0.26 | 0.31 | 0.27 | 3 |
| g__Desulfurispora | NA | NA | 0.00 | NA | 1 |
| g__Dialister | 0.01 | NA | 0.60 | 0.57 | 2 |
| g__Dielma | 0.00 | 0.00 | 0.00 | 0.00 | 3 |
| g__Dolosigranulum | NA | NA | 0.00 | NA | 1 |
| g__Dorea | 0.01 | 0.42 | 1.09 | 1.37 | 3 |
| g__Dyadobacter | NA | NA | 0.00 | NA | 1 |
| g__Dysgonomonas | NA | 0.00 | 0.00 | 0.00 | 3 |
| g__Eggerthella | 0.00 | NA | 0.00 | 0.00 | 2 |
| g__Eggerthia | NA | NA | 0.00 | NA | 1 |
| g__Eikenella | NA | NA | 0.00 | 0.00 | 2 |
| g__Elusimicrobium | 0.00 | 0.00 | 0.00 | 0.00 | 3 |
| g__Emticicia | NA | 0.00 | NA | NA | 1 |
| g__Enhydrobacter | NA | NA | NA | 0.00 | 1 |
| g__Enorma | 0.00 | NA | NA | NA | 0 |
| g__Enterobacter | 0.00 | 0.00 | 0.58 | NA | 2 |
| g__Enterobacteriaceae | 0.01 | NA | NA | NA | 0 |
| g__Enterococcus | 0.00 | 0.00 | 0.22 | 0.17 | 3 |
| g__Enterorhabdus | 0.00 | NA | 0.00 | 0.00 | 2 |
| g__Epulopiscium | NA | NA | 0.04 | 0.03 | 2 |
| g__Erwinia | NA | NA | 0.00 | NA | 1 |
| g__Erysipelatoclostridium | 0.00 | 0.01 | 0.06 | 0.06 | 3 |
| g__Erysipelotrichaceae | 0.00 | NA | 1.12 | 0.94 | 2 |
| g__Escherichia | 0.00 | 0.01 | 0.10 | 0.97 | 3 |
| g__Ethanoligenens | NA | 0.00 | 0.00 | NA | 2 |
| g__Eubacterium | 0.05 | 0.00 | 5.21 | 0.02 | 3 |
| g__Ewingella | NA | NA | 0.00 | NA | 1 |
| g__Exiguobacterium | NA | NA | 0.00 | NA | 1 |
| g__Facklamia | NA | NA | 0.00 | NA | 1 |
| g__Faecalibacterium | 0.04 | 2.81 | 5.51 | 4.51 | 3 |
| g__Faecalicoccus | 0.00 | 0.00 | 0.00 | 0.00 | 3 |
| g__Faecalitalea | 0.00 | 0.01 | 0.01 | 0.02 | 3 |
| g__Falsirhodobacter | NA | NA | 0.00 | NA | 1 |
| g__Fenollaria | 0.00 | NA | NA | NA | 0 |
| g__Fibrobacter | NA | NA | 0.00 | NA | 1 |
| g__Filifactor | NA | 0.00 | 0.00 | 0.00 | 3 |
| g__Finegoldia | 0.00 | NA | 0.00 | 0.00 | 2 |
| g__Firmicutes | 0.00 | NA | NA | NA | 0 |
| g__Flavobacterium | NA | NA | 0.00 | NA | 1 |
| g__Flavonifractor | 0.00 | NA | 0.05 | 0.07 | 2 |
| g__Fluviicola | NA | NA | 0.00 | NA | 1 |
| g__Fructobacillus | NA | NA | 0.00 | 0.00 | 2 |
| g__Fusobacterium | 0.00 | 0.07 | 0.14 | 0.12 | 3 |
| g__Gardnerella | 0.00 | NA | 0.00 | 0.00 | 2 |
| g__Gemella | 0.00 | NA | 0.03 | 0.03 | 2 |

| | | | | | |
|---|---|---|---|---|---|
| g__Geminicoccus | NA | NA | 0.00 | NA | 1 |
| g__Gemmobacter | NA | NA | 0.00 | NA | 1 |
| g__Geobacillus | NA | NA | 0.00 | NA | 1 |
| g__Geodermatophilus | NA | NA | 0.01 | 0.01 | 2 |
| g__Glutamicibacter | NA | 0.00 | NA | NA | 1 |
| g__Granulicatella | 0.00 | 0.00 | 0.00 | 0.01 | 3 |
| g__Haemophilus | 0.00 | 0.00 | 0.00 | 0.03 | 3 |
| g__Hafnia | 0.00 | NA | 0.00 | NA | 1 |
| g__Helcococcus | NA | NA | 0.00 | NA | 1 |
| g__Helicobacter | 0.00 | NA | NA | NA | 0 |
| g__Herbaspirillum | NA | NA | 0.00 | NA | 1 |
| g__Holdemanella | 0.00 | 0.14 | 0.18 | 0.26 | 3 |
| g__Holdemania | 0.00 | 0.00 | 0.01 | 0.01 | 3 |
| g__Hungatella | 0.00 | NA | 0.01 | 0.01 | 2 |
| g__Hymenobacter | NA | NA | 0.00 | 0.00 | 2 |
| g__Hyphomicrobium | NA | NA | 0.00 | NA | 1 |
| g__Intestinibacter | 0.00 | 0.00 | 0.02 | 0.27 | 3 |
| g__Intestinimonas | 0.00 | NA | 0.01 | 0.13 | 2 |
| g__Isoptericola | NA | NA | 0.00 | NA | 1 |
| g__Janibacter | NA | NA | 0.00 | 0.00 | 2 |
| g__Janthinobacterium | NA | NA | 0.00 | NA | 1 |
| g__Johnsonella | NA | 0.00 | 0.00 | 0.00 | 3 |
| g__Kitasatospora | NA | NA | 0.00 | NA | 1 |
| g__Klebsiella | 0.00 | 0.00 | 0.00 | 0.02 | 3 |
| g__Kluyvera | NA | 0.00 | 0.00 | NA | 2 |
| g__Knoellia | NA | NA | 0.00 | NA | 1 |
| g__Kocuria | NA | NA | 0.00 | 0.00 | 2 |
| g__Kosakonia | NA | 0.00 | NA | NA | 1 |
| g__Kurthia | NA | NA | 0.00 | NA | 1 |
| g__Lachnoanaerobaculum | 0.00 | 0.00 | 0.00 | 0.00 | 3 |
| g__Lachnoclostridium | 0.00 | 1.36 | 2.73 | 1.23 | 3 |
| g__Lachnospira | NA | 0.77 | 0.76 | 1.63 | 3 |
| g__Lachnospiraceae | 0.02 | NA | 2.87 | 1.91 | 2 |
| g__Lacticigenium | NA | NA | 0.00 | NA | 1 |
| g__Lactobacillus | 0.00 | 1.36 | 0.19 | 0.26 | 3 |
| g__Lactococcus | 0.00 | 0.19 | 0.23 | 0.19 | 3 |
| g__Lautropia | 0.00 | NA | NA | 0.00 | 1 |
| g__Lawsonella | NA | NA | NA | 0.00 | 1 |
| g__Legionella | NA | NA | 0.00 | NA | 1 |
| g__Leifsonia | NA | NA | 0.00 | NA | 1 |
| g__Leminorella | NA | NA | 0.00 | NA | 1 |
| g__Leptothrix | NA | NA | 0.00 | NA | 1 |
| g__Leptotrichia | NA | 0.00 | 0.00 | 0.00 | 3 |
| g__Leucobacter | NA | NA | 0.00 | 0.00 | 2 |
| g__Leuconostoc | 0.00 | 0.02 | 0.02 | 0.02 | 3 |
| g__Levyella | 0.00 | NA | NA | NA | 0 |
| g__Libanicoccus | 0.00 | NA | NA | 0.00 | 1 |
| g__Listeria | NA | NA | 0.00 | NA | 1 |
| g__Luteibacter | NA | NA | 0.00 | NA | 1 |
| g__Lysinibacillus | NA | NA | 0.00 | 0.00 | 2 |
| g__Lysobacter | NA | NA | 0.00 | NA | 1 |
| g__Macrococcus | 0.00 | NA | 0.00 | NA | 1 |
| g__Mailhella | 0.00 | NA | NA | 0.02 | 1 |
| g__Mannheimia | NA | NA | 0.00 | 0.00 | 2 |
| g__Marinobacter | NA | NA | 0.00 | NA | 1 |

| | | | | | |
|---|---|---|---|---|---|
| g__Marinococcus | NA | NA | 0.00 | NA | 1 |
| g__Marinomonas | NA | NA | 0.00 | NA | 1 |
| g__Marmoricola | NA | NA | 0.00 | NA | 1 |
| g__Marvinbryantia | NA | 0.05 | 0.85 | 0.13 | 3 |
| g__Massilia | NA | NA | 0.00 | NA | 1 |
| g__Massiliomicrobiota | 0.00 | NA | NA | NA | 0 |
| g__Megamonas | 0.00 | NA | 0.39 | 0.34 | 2 |
| g__Megasphaera | 0.00 | NA | 0.66 | 0.68 | 2 |
| g__Melissococcus | NA | NA | 0.00 | NA | 1 |
| g__Merdibacter | 0.00 | NA | NA | 0.00 | 1 |
| g__Mesorhizobium | NA | NA | 0.00 | NA | 1 |
| g__Methanobacterium | NA | 0.00 | 0.00 | NA | 2 |
| g__Methanobrevibacter | 0.01 | 0.69 | 1.08 | 0.93 | 3 |
| g__Methanomassiliicoccus | NA | NA | 0.00 | NA | 1 |
| g__Methanosphaera | 0.00 | 0.01 | 0.06 | 0.06 | 3 |
| g__Methylobacillus | NA | 0.00 | 0.00 | 0.00 | 3 |
| g__Methylobacterium | NA | 0.00 | 0.01 | 0.00 | 3 |
| g__Methylovorus | NA | NA | 0.00 | NA | 1 |
| g__Microbacterium | 0.00 | NA | 0.00 | NA | 1 |
| g__Micrococcus | NA | NA | 0.00 | 0.00 | 2 |
| g__Microvirga | NA | NA | NA | 0.00 | 1 |
| g__Mitsuokella | 0.00 | NA | 0.17 | 0.13 | 2 |
| g__Mobiluncus | NA | NA | 0.00 | NA | 1 |
| g__Modestobacter | NA | NA | 0.00 | NA | 1 |
| g__Mogibacterium | 0.00 | 0.07 | 0.08 | 0.07 | 3 |
| g__Moraxella | NA | NA | 0.00 | NA | 1 |
| g__Morganella | NA | NA | 0.00 | NA | 1 |
| g__Mucilaginibacter | NA | NA | NA | 0.00 | 1 |
| g__Mucispirillum | NA | NA | 0.00 | NA | 1 |
| g__Mycetocola | NA | NA | 0.00 | NA | 1 |
| g__Mycobacterium | NA | NA | 0.00 | NA | 1 |
| g__Mycoplasma | 0.00 | NA | NA | NA | 0 |
| g__Neglecta | 0.00 | NA | NA | NA | 0 |
| g__Neisseria | NA | 0.00 | 0.00 | 0.00 | 3 |
| g__Nesterenkonia | NA | NA | 0.00 | 0.00 | 2 |
| g__Niabella | NA | NA | 0.00 | NA | 1 |
| g__Niameybacter | 0.00 | NA | NA | NA | 0 |
| g__Nitrobacter | NA | NA | 0.00 | NA | 1 |
| g__Nocardioides | NA | NA | 0.00 | 0.00 | 2 |
| g__Novosphingobium | NA | NA | 0.00 | 0.00 | 2 |
| g__Oceanobacillus | NA | NA | 0.00 | NA | 1 |
| g__Ochrobactrum | NA | NA | 0.00 | NA | 1 |
| g__Odoribacter | 0.00 | NA | 0.09 | 0.09 | 2 |
| g__Olsenella | 0.00 | NA | 0.00 | 0.00 | 2 |
| g__Oribacterium | 0.00 | 0.00 | 0.00 | 0.00 | 3 |
| g__Ornithinibacillus | NA | NA | 0.00 | NA | 1 |
| g__Oscillibacter | 0.01 | NA | 0.02 | 0.14 | 2 |
| g__Ottowia | NA | NA | 0.00 | 0.00 | 2 |
| g__Oxalobacter | NA | NA | 0.01 | 0.01 | 2 |
| g__Paenibacillus | NA | NA | 0.00 | NA | 1 |
| g__Paeniclostridium | NA | 0.00 | NA | 0.00 | 2 |
| g__Paenirhodobacter | NA | NA | 0.00 | NA | 1 |
| g__Paenisporosarcina | NA | NA | 0.00 | NA | 1 |
| g__Pantoea | NA | NA | 0.00 | 0.00 | 2 |
| g__Parabacteroides | 0.00 | 0.58 | 0.45 | 0.41 | 3 |

| | | | | | |
|---|---|---|---|---|---|
| g__Paraclostridium | NA | 0.00 | NA | NA | 1 |
| g__Paracoccus | NA | 1.77 | NA | 1.96 | 2 |
| g__Paraprevotella | 0.00 | 0.28 | 0.29 | 0.27 | 3 |
| g__Parascardovia | 0.00 | NA | 0.00 | NA | 1 |
| g__Parasutterella | 0.00 | NA | 0.00 | 0.20 | 2 |
| g__Parvimonas | 0.00 | NA | 0.12 | 0.10 | 2 |
| g__Pasteurella | NA | NA | 0.00 | NA | 1 |
| g__Patulibacter | NA | NA | 0.00 | NA | 1 |
| g__Pectobacterium | NA | NA | 0.00 | NA | 1 |
| g__Pediococcus | 0.00 | 0.00 | 0.00 | 0.00 | 3 |
| g__Pedobacter | NA | 0.00 | 0.00 | NA | 2 |
| g__Peptoclostridium | NA | 0.00 | 0.12 | NA | 2 |
| g__Peptoniphilus | 0.00 | NA | 0.01 | 0.01 | 2 |
| g__Peptostreptococcaceae | 0.00 | NA | NA | NA | 0 |
| g__Peptostreptococcus | 0.00 | 0.00 | 0.05 | 0.04 | 3 |
| g__Phascolarctobacterium | 0.01 | NA | 1.05 | 1.30 | 2 |
| g__Phenylobacterium | NA | NA | 0.00 | NA | 1 |
| g__Phocaeicola | NA | NA | 0.00 | 0.00 | 2 |
| g__Photorhabdus | NA | NA | 0.00 | NA | 1 |
| g__Phyllobacterium | NA | NA | 0.00 | 0.00 | 2 |
| g__Planococcus | NA | 0.00 | 0.00 | NA | 2 |
| g__Planomicrobium | NA | NA | 0.01 | NA | 1 |
| g__Pleomorphomonas | NA | NA | 0.00 | NA | 1 |
| g__Polaromonas | NA | NA | 0.00 | NA | 1 |
| g__Porphyromonadaceae | 0.00 | NA | NA | NA | 0 |
| g__Porphyromonas | 0.00 | 0.00 | 0.00 | 0.00 | 3 |
| g__Prevotella | 0.05 | 0.11 | 4.15 | 5.28 | 3 |
| g__Prevotellaceae | NA | NA | 0.11 | 0.10 | 2 |
| g__Prochlorococcus | NA | NA | 0.00 | NA | 1 |
| g__Propionibacterium | 0.00 | NA | 0.00 | NA | 1 |
| g__Proteiniclasticum | NA | NA | 0.00 | NA | 1 |
| g__Proteus | NA | NA | 0.00 | NA | 1 |
| g__Providencia | NA | NA | 0.00 | NA | 1 |
| g__Pseudobutyrivibrio | NA | 0.01 | 1.76 | NA | 2 |
| g__Pseudoclavibacter | NA | NA | 0.00 | NA | 1 |
| g__Pseudoflavonifractor | 0.00 | NA | NA | 0.00 | 1 |
| g__Pseudomonas | 0.00 | 0.00 | 0.25 | 0.00 | 3 |
| g__Pseudoramibacter | NA | NA | 0.00 | 0.00 | 2 |
| g__Pseudoxanthomonas | NA | 0.00 | 0.00 | NA | 2 |
| g__Psychrobacter | NA | 0.00 | 0.00 | NA | 2 |
| g__Pyramidobacter | 0.00 | 0.00 | 0.00 | 0.00 | 3 |
| g__Rahnella | NA | NA | 0.00 | NA | 1 |
| g__Ralstonia | NA | NA | 0.00 | NA | 1 |
| g__Raoultella | 0.00 | NA | 0.00 | 0.00 | 2 |
| g__Rheinheimera | NA | NA | 0.00 | NA | 1 |
| g__Rhizobium | NA | NA | 0.00 | NA | 1 |
| g__Rhodobacter | NA | NA | 0.00 | NA | 1 |
| g__Rhodococcus | NA | NA | 0.00 | NA | 1 |
| g__Rhodopseudomonas | NA | 0.00 | 0.00 | 0.00 | 3 |
| g__Rhodovulum | NA | NA | 0.00 | NA | 1 |
| g__Rikenella | NA | 0.00 | NA | 0.00 | 2 |
| g__Robinsoniella | NA | 0.00 | 0.00 | 0.00 | 3 |
| g__Roseburia | 0.02 | 2.35 | 1.69 | 0.81 | 3 |
| g__Roseobacter | NA | NA | 0.00 | NA | 1 |
| g__Roseomonas | NA | NA | 0.00 | 0.00 | 2 |

| | | | | | |
|---|---|---|---|---|---|
| g__Roseovarius | NA | NA | 0.00 | NA | 1 |
| g__Rothia | 0.00 | 0.01 | 0.01 | 0.01 | 3 |
| g__Rubellimicrobium | NA | NA | 0.00 | 0.00 | 2 |
| g__Rubrivivax | NA | NA | 0.00 | NA | 1 |
| g__Rubrobacter | NA | NA | 0.00 | 0.00 | 2 |
| g__Ruminiclostridium | 0.01 | 0.08 | 1.44 | 0.79 | 3 |
| g__Ruminococcaceae | 0.00 | NA | 5.54 | 5.94 | 2 |
| g__Ruminococcus | 0.08 | 5.25 | 7.03 | 7.55 | 3 |
| g__Salmonella | NA | 0.00 | 0.00 | NA | 2 |
| g__Scardovia | 0.00 | NA | 0.00 | 0.00 | 2 |
| g__Segetibacter | NA | NA | 0.00 | NA | 1 |
| g__Selenomonas | 0.00 | NA | 0.00 | 0.01 | 2 |
| g__Senegalimassilia | 0.00 | NA | 0.00 | 0.00 | 2 |
| g__Serratia | 0.00 | NA | 0.07 | NA | 1 |
| g__Shewanella | NA | NA | 0.00 | NA | 1 |
| g__Shimwellia | NA | 0.00 | 0.00 | NA | 2 |
| g__Shuttleworthia | NA | NA | 0.00 | 0.01 | 2 |
| g__Simplicispira | NA | NA | 0.00 | NA | 1 |
| g__Slackia | 0.00 | NA | 0.00 | NA | 1 |
| g__Smithella | NA | NA | 0.00 | NA | 1 |
| g__Sodalis | NA | NA | 0.00 | NA | 1 |
| g__Solibacillus | 0.00 | NA | 0.00 | NA | 1 |
| g__Solobacterium | 0.00 | 0.02 | 0.03 | 0.02 | 3 |
| g__Sphingobacterium | NA | NA | 0.00 | NA | 1 |
| g__Sphingobium | NA | NA | 0.00 | NA | 1 |
| g__Sphingomonas | NA | 4.69 | 4.05 | 4.97 | 3 |
| g__Sphingopyxis | NA | NA | 0.00 | NA | 1 |
| g__Spirosoma | NA | NA | 0.00 | 0.00 | 2 |
| g__Sporosarcina | 0.00 | 0.00 | 0.00 | NA | 2 |
| g__Staphylococcus | 0.00 | 0.00 | 0.01 | 0.01 | 3 |
| g__Stenotrophomonas | NA | NA | 0.00 | 0.00 | 2 |
| g__Stomatobaculum | 0.00 | NA | 0.00 | 0.00 | 2 |
| g__Streptococcus | 0.03 | 3.24 | 4.44 | 4.04 | 3 |
| g__Streptomyces | NA | NA | 0.00 | NA | 1 |
| g__Subdoligranulum | 0.00 | 2.40 | 2.09 | 2.30 | 3 |
| g__Succinatimonas | 0.00 | 0.00 | 0.01 | 0.01 | 3 |
| g__Succinivibrio | 0.00 | 0.24 | 0.00 | 0.25 | 3 |
| g__Sulfitobacter | NA | 6.77 | NA | NA | 1 |
| g__Sutterella | 0.00 | NA | 0.20 | 0.15 | 2 |
| g__Synergistes | 0.00 | 0.00 | 0.00 | 0.00 | 3 |
| g__Tatumella | NA | NA | 0.00 | NA | 1 |
| g__Terrisporobacter | 0.00 | NA | 0.04 | 0.06 | 2 |
| g__Tetragenococcus | NA | NA | 0.00 | NA | 1 |
| g__Tetrasphaera | NA | NA | 0.00 | NA | 1 |
| g__Thalassospira | NA | NA | 0.06 | NA | 1 |
| g__Thauera | NA | NA | 0.00 | NA | 1 |
| g__Thermicanus | NA | NA | 0.00 | NA | 1 |
| g__Tissierellia | 0.00 | NA | NA | NA | 0 |
| g__Trabulsiella | NA | NA | 0.00 | NA | 1 |
| g__Treponema | NA | 0.00 | 0.00 | 0.00 | 3 |
| g__Tropheryma | 0.00 | NA | 0.00 | NA | 1 |
| g__Truepera | NA | NA | 0.00 | 0.00 | 2 |
| g__Turicibacter | 0.00 | 0.05 | 0.00 | 0.06 | 3 |
| g__Tyzzerella | 0.00 | 0.06 | 0.07 | 0.12 | 3 |
| g__unknown | 0.02 | NA | NA | NA | 0 |

| | | | | | |
|---|---|---|---|---|---|
| g__unknown Atopobiaceae | 0.00 | NA | NA | NA | 0 |
| g__unknown Bacillales | 0.00 | NA | NA | NA | 0 |
| g__unknown Bacilli | 0.00 | NA | NA | NA | 0 |
| g__unknown Bacteria | 0.00 | NA | NA | NA | 0 |
| g__unknown Bacteroidaceae | 0.00 | NA | NA | NA | 0 |
| g__unknown Bacteroidales | 0.01 | NA | NA | NA | 0 |
| g__unknown Burkholderiales | 0.00 | NA | NA | NA | 0 |
| g__unknown candidatus Saccharibacteria | 0.00 | NA | NA | NA | 0 |
| g__unknown cellular organisms | 0.00 | NA | NA | NA | 0 |
| g__unknown Clostridia | 0.00 | NA | NA | NA | 0 |
| g__unknown Clostridiaceae | 0.00 | NA | NA | NA | 0 |
| g__unknown Clostridiales | 0.13 | NA | NA | NA | 0 |
| g__unknown Clostridiales Family XIII. Incertae : | 0.00 | NA | NA | NA | 0 |
| g__unknown Comamonadaceae | 0.00 | NA | NA | NA | 0 |
| g__unknown Dehalococcoidales | 0.00 | NA | NA | NA | 0 |
| g__unknown Eggerthellaceae | 0.00 | NA | NA | NA | 0 |
| g__unknown Eggerthellales | 0.00 | NA | NA | NA | 0 |
| g__unknown Enterobacterales | 0.00 | NA | NA | NA | 0 |
| g__unknown Erysipelotrichaceae | 0.00 | NA | NA | NA | 0 |
| g__unknown Firmicutes | 0.03 | NA | NA | NA | 0 |
| g__unknown Flavobacteriia | 0.00 | NA | NA | NA | 0 |
| g__unknown Lachnospiraceae | 0.01 | NA | NA | NA | 0 |
| g__unknown Lactobacillales | 0.00 | NA | NA | NA | 0 |
| g__unknown Lentisphaerae | 0.00 | NA | NA | NA | 0 |
| g__unknown Methanomicrobia | 0.00 | NA | NA | NA | 0 |
| g__unknown Pasteurellaceae | 0.00 | NA | NA | NA | 0 |
| g__unknown Peptostreptococcaceae | 0.00 | NA | NA | NA | 0 |
| g__unknown Prevotellaceae | 0.00 | NA | NA | NA | 0 |
| g__unknown Rhodospirillaceae | 0.00 | NA | NA | NA | 0 |
| g__unknown Ruminococcaceae | 0.01 | NA | NA | NA | 0 |
| g__unknown Sutterellaceae | 0.00 | NA | NA | NA | 0 |
| g__unknown Synergistaceae | 0.00 | NA | NA | NA | 0 |
| g__unknown Tenericutes | 0.00 | NA | NA | NA | 0 |
| g__unknown Veillonellaceae | 0.00 | NA | NA | NA | 0 |
| g__unknown Verrucomicrobia | 0.00 | NA | NA | NA | 0 |
| g__Ureibacillus | NA | NA | 0.00 | NA | 1 |
| g__Vagococcus | NA | NA | 0.00 | NA | 1 |
| g__Varibaculum | 0.00 | 0.00 | 0.00 | 0.00 | 3 |
| g__Variovorax | NA | 0.00 | 0.00 | 0.00 | 3 |
| g__Veillonella | 0.00 | NA | 0.10 | 0.10 | 2 |
| g__Vibrio | NA | 0.00 | 0.00 | 0.00 | 3 |
| g__Virgibacillus | NA | NA | 0.00 | NA | 1 |
| g__Weissella | 0.00 | 0.00 | 0.00 | 0.00 | 3 |
| g__Xanthobacter | NA | NA | 0.00 | NA | 1 |
| g__Xanthomonas | NA | NA | 0.00 | NA | 1 |
| g__Xenorhabdus | NA | NA | 0.00 | NA | 1 |
| g__Yersinia | NA | NA | 0.00 | NA | 1 |
| 196 | NA | NA | 0.00 | NA | 1 |
| 306 | NA | NA | 0.00 | NA | 1 |
| 822 | NA | NA | 0.00 | NA | 1 |
| g__ | NA | NA | 0.24 | NA | 1 |
| g__28-4 | NA | NA | NA | 0.03 | 1 |
| g__Acetanaerobacterium | NA | 0.00 | 0.00 | 0.01 | 3 |
| g__Acetatifactor | NA | 0.00 | NA | NA | 1 |
| g__Acetitomaculum | NA | 0.00 | 0.01 | NA | 2 |

| | | | | | |
|---|---|---|---|---|---|
| g__Aeribacillus | NA | NA | 0.00 | NA | 1 |
| g__Aeriscardovia | NA | NA | 0.00 | NA | 1 |
| g__Agathobacter | NA | NA | NA | 1.63 | 1 |
| g__Albidovulum | NA | NA | 0.00 | NA | 1 |
| g__Allisonella | NA | NA | 0.01 | 0.01 | 2 |
| g__alpha-proteobacterium | NA | NA | 0.00 | NA | 1 |
| g__Altererythrobacter | NA | NA | 0.00 | NA | 1 |
| g__Amaricoccus | NA | NA | 0.00 | NA | 1 |
| g__Anaerofilum | NA | 0.01 | 0.01 | 0.05 | 3 |
| g__Anaeroplasma | NA | 0.00 | NA | 0.09 | 2 |
| g__Anaerosporobacter | NA | 0.03 | 0.00 | NA | 2 |
| g__Angelakisella | NA | NA | NA | 0.02 | 1 |
| g__Arabidopsis | NA | NA | 0.00 | NA | 1 |
| g__Artemisia | NA | NA | 0.00 | NA | 1 |
| g__Asaccharospora | NA | NA | 0.00 | 0.00 | 2 |
| g__Asteroleplasma | NA | NA | 0.00 | 0.00 | 2 |
| g__Atlantibacter | NA | NA | NA | 0.00 | 1 |
| g__bacterium | NA | NA | 0.00 | NA | 1 |
| g__Bergeriella | NA | NA | 0.00 | NA | 1 |
| g__Blastocatella | NA | 0.00 | 0.00 | 0.00 | 3 |
| g__Blastocystis | NA | NA | 0.00 | NA | 1 |
| g__CAG-352 | NA | NA | NA | 0.21 | 1 |
| g__CAG-56 | NA | NA | NA | 0.12 | 1 |
| g__CAG-873 | NA | NA | NA | 0.00 | 1 |
| g__Candidatus | NA | NA | 0.01 | NA | 1 |
| g__Candidatus_Soleaferrea | NA | 0.00 | NA | 0.01 | 2 |
| g__Candidatus_Stoquefichus | NA | NA | NA | 0.00 | 1 |
| g__Caproiciproducens | NA | 0.04 | NA | 0.01 | 2 |
| g__Carica | NA | NA | 0.00 | NA | 1 |
| g__Caryophanon | NA | NA | 0.00 | NA | 1 |
| g__Catabacter | NA | NA | 0.00 | 0.00 | 2 |
| g__Catenibacterium | NA | 0.00 | 0.27 | 0.22 | 3 |
| g__Catenisphaera | NA | 0.07 | NA | 0.13 | 2 |
| g__CENA359 | NA | NA | NA | 0.00 | 1 |
| g__Cerasicoccus | NA | 0.00 | NA | NA | 1 |
| g__CHKCI002 | NA | NA | NA | 0.00 | 1 |
| g__Chlorochromatium | NA | NA | 0.00 | NA | 1 |
| g__Christensenella | NA | 0.00 | 0.00 | 0.89 | 3 |
| g__Christensenellaceae | NA | NA | 1.38 | NA | 1 |
| g__Chryseomicrobium | NA | NA | 0.00 | NA | 1 |
| g__Chungangia | NA | NA | 0.00 | NA | 1 |
| g__Cloacibacillus | NA | 0.04 | 0.07 | 0.05 | 3 |
| g__Cloacibacterium | NA | NA | 0.00 | 0.00 | 2 |
| g__Clostridiales-bacterium | NA | NA | 0.00 | NA | 1 |
| g__Coprobacter | NA | 0.03 | 0.03 | 0.04 | 3 |
| g__Coriobacteriaceae_UCG | NA | NA | 0.00 | 0.00 | 2 |
| g__Cuneatibacter | NA | NA | NA | 0.00 | 1 |
| g__Defluviitaleaceae | NA | NA | 0.04 | 0.02 | 2 |
| g__Domibacillus | NA | NA | 0.00 | NA | 1 |
| g__Donghicola | NA | NA | 0.00 | NA | 1 |
| g__DTU089 | NA | NA | NA | 0.01 | 1 |
| g__Eisenbergiella | NA | 0.03 | 0.01 | 0.05 | 3 |
| g__Eutrema | NA | NA | 0.00 | NA | 1 |
| g__Extensimonas | NA | NA | 0.00 | 0.00 | 2 |
| g__Ezakiella | NA | NA | 0.00 | 0.00 | 2 |

| | | | | | |
|---|---|---|---|---|---|
| g__F0332 | NA | NA | NA | 0.00 | 1 |
| g__Family | NA | NA | 0.28 | NA | 1 |
| g__Family_XIII_AD3011_group | NA | NA | NA | 0.24 | 1 |
| g__Family_XIII_UCG-001 | NA | NA | NA | 0.04 | 1 |
| g__Fastidiosipila | NA | 0.00 | 0.00 | NA | 2 |
| g__Flavisolibacter | NA | NA | NA | 0.00 | 1 |
| g__Fournierella | NA | NA | NA | 0.03 | 1 |
| g__Fretibacterium | NA | 0.00 | 0.00 | 0.00 | 3 |
| g__Frigoribacterium | NA | NA | 0.00 | NA | 1 |
| g__Fusicatenibacter | NA | 0.65 | 0.88 | 0.67 | 3 |
| g__Gallicola | NA | NA | 0.00 | NA | 1 |
| g__GCA-900066225 | NA | NA | NA | 0.04 | 1 |
| g__GCA-900066575 | NA | NA | NA | 0.05 | 1 |
| g__GCA-900066755 | NA | NA | NA | 0.00 | 1 |
| g__Gelria | NA | 0.00 | 0.00 | NA | 2 |
| g__Georgenia | NA | NA | 0.00 | NA | 1 |
| g__Gordonibacter | NA | NA | 0.00 | 0.00 | 2 |
| g__Hafnia-Obesumbacterium | NA | NA | NA | 0.05 | 1 |
| g__Harryflintia | NA | NA | NA | 0.00 | 1 |
| g__Hedyosmum | NA | NA | 0.00 | NA | 1 |
| g__Herbinix | NA | NA | NA | 0.00 | 1 |
| g__Hespellia | NA | NA | 0.00 | NA | 1 |
| g__Howardella | NA | NA | 0.05 | 0.06 | 2 |
| g__Hydrogenoanaerobacterium | NA | NA | 0.00 | 0.00 | 2 |
| g__Ideonella | NA | NA | 0.00 | NA | 1 |
| g__Incertae | NA | NA | 1.64 | NA | 1 |
| g__Isobaculum | NA | NA | 0.00 | NA | 1 |
| g__Jeotgalibacillus | NA | NA | 0.00 | NA | 1 |
| g__Lactonifactor | NA | NA | 0.02 | 0.00 | 2 |
| g__Lupinus | NA | NA | 0.00 | NA | 1 |
| g__Lutispora | NA | NA | 0.00 | NA | 1 |
| g__Marinilactibacillus | NA | NA | 0.00 | NA | 1 |
| g__Marinovum | NA | NA | 0.00 | NA | 1 |
| g__Metascardovia | NA | NA | 0.00 | NA | 1 |
| g__methanogenic | NA | NA | 0.01 | NA | 1 |
| g__Microbacter | NA | NA | 0.00 | NA | 1 |
| g__Mobilitalea | NA | 0.00 | 0.00 | NA | 2 |
| g__Moryella | NA | 0.08 | 0.01 | 0.00 | 3 |
| g__Murdochiella | NA | NA | 0.00 | 0.00 | 2 |
| g__Musa | NA | NA | 0.00 | NA | 1 |
| g__NA | NA | 42.27 | NA | 10.60 | 2 |
| g__Natranaerovirga | NA | NA | 0.00 | NA | 1 |
| g__Negativibacillus | NA | NA | NA | 0.11 | 1 |
| g__Negativicoccus | NA | NA | 0.00 | NA | 1 |
| g__Noviherbaspirillum | NA | NA | 0.00 | NA | 1 |
| g__Oenothera | NA | NA | 0.00 | NA | 1 |
| g__Oscillospira | NA | 0.20 | 0.01 | 0.02 | 3 |
| g__Paenalcaligenes | NA | NA | 0.00 | NA | 1 |
| g__Panax | NA | NA | 0.00 | NA | 1 |
| g__Pantanalinema_CENA516 | NA | NA | NA | 0.00 | 1 |
| g__Papillibacter | NA | 0.01 | 0.00 | 0.00 | 3 |
| g__Paracocccus | NA | NA | 0.03 | NA | 1 |
| g__Paraeggerthella | NA | NA | 0.00 | NA | 1 |
| g__Paraherbaspirillum | NA | NA | NA | 0.00 | 1 |
| g__Parasporobacterium | NA | NA | 0.00 | NA | 1 |

| | | | | | |
|---|---|---|---|---|---|
| g__Parvibacter | NA | NA | 0.00 | NA | 1 |
| g__Paucibacter | NA | NA | 0.00 | NA | 1 |
| g__Paucimonas | NA | NA | 0.00 | NA | 1 |
| g__Pectinatus | NA | NA | 0.00 | NA | 1 |
| g__Pelomonas | NA | NA | 0.00 | NA | 1 |
| g__Pelospora | NA | NA | NA | 0.00 | 1 |
| g__Peptococcus | NA | 0.00 | 0.05 | 0.03 | 3 |
| g__Phocea | NA | NA | NA | 0.00 | 1 |
| g__Phreatobacter | NA | NA | 0.00 | NA | 1 |
| g__Phytophthora | NA | NA | 0.00 | NA | 1 |
| g__Plesiomonas | NA | NA | 0.00 | NA | 1 |
| g__PMMR1 | NA | NA | NA | 0.00 | 1 |
| g__Porphyromonadaceae-bacterium | NA | NA | 0.00 | NA | 1 |
| g__possible | NA | NA | 0.00 | NA | 1 |
| g__Pragia | NA | NA | 0.00 | NA | 1 |
| g__Prunus | NA | NA | 0.00 | NA | 1 |
| g__Pseudacidovorax | NA | NA | 0.00 | NA | 1 |
| g__Pseudochrobactrum | NA | NA | 0.00 | NA | 1 |
| g__Pseudoscardovia | NA | NA | 0.00 | NA | 1 |
| g__Psychrobacillus | NA | NA | 0.00 | NA | 1 |
| g__Pygmaiobacter | NA | NA | NA | 0.00 | 1 |
| g__Raoultibacter | NA | NA | NA | 0.00 | 1 |
| g__Rarobacter | NA | NA | 0.00 | NA | 1 |
| g__RB41 | NA | NA | NA | 0.00 | 1 |
| g__Rikenellaceae | NA | NA | 0.08 | 0.05 | 2 |
| g__Romboutsia | NA | NA | 0.58 | 0.54 | 2 |
| g__Rosenbergiella | NA | 0.00 | NA | 0.00 | 2 |
| g__Roseococcus | NA | NA | 0.00 | NA | 1 |
| g__Rubrivirga | NA | NA | NA | 0.00 | 1 |
| g__Salinihabitans | NA | NA | 0.00 | NA | 1 |
| g__Sanguibacteroides | NA | NA | NA | 0.00 | 1 |
| g__Sarcina | NA | NA | 0.08 | NA | 1 |
| g__Schwartzia | NA | NA | 0.00 | NA | 1 |
| g__Scytonema_UTEX_2349 | NA | NA | NA | 0.00 | 1 |
| g__Sellimonas | NA | 0.02 | NA | 0.14 | 2 |
| g__Shigella | NA | 0.00 | NA | NA | 1 |
| g__Solirubrobacter | NA | NA | 0.00 | NA | 1 |
| g__Sphingoaurantiacus | NA | NA | NA | 0.00 | 1 |
| g__Sphingomonadaceae-bacterium | NA | NA | 0.00 | NA | 1 |
| g__Sphingosinicella | NA | NA | 0.00 | NA | 1 |
| g__Sporacetigenium | NA | NA | 0.00 | NA | 1 |
| g__Sporobacter | NA | 0.00 | 0.00 | 0.00 | 3 |
| g__Stakelama | NA | NA | 0.00 | NA | 1 |
| g__Succiniclasticum | NA | NA | 0.00 | 0.00 | 2 |
| g__Sulfurifustis | NA | 0.00 | NA | NA | 1 |
| g__Syntrophococcus | NA | 0.00 | 0.00 | 0.02 | 3 |
| g__Tepidimonas | NA | NA | 0.00 | NA | 1 |
| g__Thioprofundum | NA | 0.00 | NA | NA | 1 |
| g__Trichococcus | NA | 0.00 | 0.00 | NA | 2 |
| g__Tropicimonas | NA | NA | 0.00 | NA | 1 |
| g__UBA1819 | NA | NA | NA | 0.10 | 1 |
| g__UC5-1-2E3 | NA | NA | NA | 0.00 | 1 |
| g__uncultured | NA | NA | 1.97 | NA | 1 |
| g__uncultured-alpha-proteobacterium | NA | NA | 0.00 | NA | 1 |
| g__uncultured-archaeon | NA | NA | 0.00 | NA | 1 |

| | | | | | |
|---|---|---|---|---|---|
| g__uncultured-bacterium | NA | NA | 0.33 | NA | 1 |
| g__uncultured-Bacteroidales-bacterium | NA | NA | 0.00 | NA | 1 |
| g__uncultured-endolithic-bacterium | NA | NA | 0.00 | NA | 1 |
| g__uncultured-Enterococcaceae-bacterium | NA | NA | 0.00 | NA | 1 |
| g__uncultured-Enterococcus | NA | NA | 0.00 | NA | 1 |
| g__uncultured-Firmicutes-bacterium | NA | NA | 0.00 | NA | 1 |
| g__uncultured-Fusobacterium | NA | NA | 0.00 | NA | 1 |
| g__uncultured-Klebsiella | NA | NA | 0.00 | NA | 1 |
| g__uncultured-Lachnospira | NA | NA | 0.00 | NA | 1 |
| g__uncultured-Lactobacillales-bacterium | NA | NA | 0.00 | NA | 1 |
| g__uncultured-organism | NA | NA | 0.02 | NA | 1 |
| g__uncultured-Porphyromonadaceae-bacterium | NA | NA | 0.00 | NA | 1 |
| g__uncultured-Prevotellaceae-bacterium | NA | NA | 0.00 | NA | 1 |
| g__uncultured-rumen-bacterium | NA | NA | 0.00 | NA | 1 |
| g__uncultured-Sphingomonadaceae-bacterium | NA | NA | 0.00 | NA | 1 |
| g__uncultured-Streptococcaceae-bacterium | NA | NA | 0.00 | NA | 1 |
| g__uncultured-Thermoanaerobacterales-bacterium | NA | NA | 0.00 | NA | 1 |
| g__Undibacterium | NA | NA | 0.00 | NA | 1 |
| g__unidentified | NA | NA | 0.00 | NA | 1 |
| g__Victivallis | NA | 0.01 | 0.02 | 0.01 | 3 |
| g__Zea | NA | NA | 0.00 | NA | 1 |
| s__[Eubacterium] brachy | NA | 0.00 | NA | NA | 1 |
| s__[Eubacterium] infirmum | NA | 0.00 | NA | NA | 1 |
| s__[Eubacterium] sulci | NA | 0.00 | NA | NA | 1 |
| s__Lachnospiraceae bacterium 8_1_57FAA | NA | 0.33 | NA | NA | 1 |
| s__Lachnospiraceae bacterium 9_1_43BFAA | NA | 0.01 | NA | NA | 1 |
| s__Peptostreptococcaceae bacterium canine oral taxon 333 | NA | 0.00 | NA | NA | 1 |
| s__Peptostreptococcaceae bacterium feline oral taxon 064 | NA | 0.01 | NA | NA | 1 |
| s__Prevotellaceae bacterium DJF_CR21k6 | NA | 0.06 | NA | NA | 1 |
| s__Prevotellaceae bacterium DJF_CR25 | NA | 0.03 | NA | NA | 1 |
| s__Prevotellaceae bacterium DJF_RP17 | NA | 1.08 | NA | NA | 1 |
| s__uncultured Christensenellaceae bacterium | NA | 0.38 | NA | NA | 1 |
| s__uncultured Clostridiales Family XIII bacterium | NA | 0.01 | NA | NA | 1 |
| s__uncultured Erysipelotrichaceae bacterium | NA | 0.00 | NA | NA | 1 |
| s__uncultured Eubacteriaceae bacterium | NA | 0.00 | NA | NA | 1 |
| s__uncultured Lachnospiraceae bacterium | NA | 0.01 | NA | NA | 1 |
| s__uncultured Peptococcaceae bacterium | NA | 0.00 | NA | NA | 1 |
| s__uncultured Prevotellaceae bacterium | NA | 0.84 | NA | NA | 1 |
| s__uncultured Ruminococcaceae bacterium | NA | 0.37 | NA | NA | 1 |
