# Supplementary material for: The Limits and Avoidance of Biases in Metagenomic Analyses of Human Fecal Microbiota
Source: Microorganisms. 2020 Dec 9;8(12):1954. doi: 10.3390/microorganisms8121954 (PMC7764459; doi:10.3390/microorganisms8121954)
Supplement: Supplementary file 1 [file microorganisms-08-01954-s001.zip › Suppl-Table-S1_article-16S-supp-tableAbundance-corrected-rev-27-11-2020-README.pdf]

color legend

	51 most abundants genera according to mOTU-v2 abundances
	genera identified by mOTU-v2 (but not the 51 most abundants)
	Genera NOT identified by mOTU-v2 but at least by one 16S pipeline
SORT1	order of the genera after filtered and “cluster” (used to recover the table
Sheet “FlashAndTrim”	Initial sequence, merging and surviving sequences avec FLASH2 and T

if other sorting are done)

Trimmomatic
